# Highlights from the Inaugural HIV Reservoirs and Immune Control Conference, October 1^st^–4^th^ 2023, Malahide Ireland

**DOI:** 10.20411/pai.v8i1.653

**Published:** 2023-12-19

**Authors:** Una O'Doherty, Javier Martinez-Picado, Asier Sáez-Cirión

**Affiliations:** 1 Department of Pathology and Laboratory Medicine, University of Pennsylvania, Philadelphia, PA; 2 IrsiCaixa AIDS Research Institute, Badalona, Spain; 3 CIBERINFEC, Instituto de Salud Carlos III, Madrid, Spain; 4 University of Vic-Central University of Catalonia (UVic-UCC), Vic, Spain; 5 Catalan Institution for Research and Advanced Studies (ICREA), Barcelona, Spain; 6 Institut Pasteur, Université Paris Cité, Unité HIV Inflammation et Persistance, Paris, France

**Keywords:** HIV-1, HIV latency, antiretroviral therapy, HIV pathogenesis, pediatric HIV infection, elite controllers, viral expression, HIV-2, innate immune responses

## Abstract

The inaugural FASEB HIV Reservoirs and Immune Control Conference brought researchers together from across the globe to discuss reservoir dynamics in clinical cohorts. It extended over 4 days in the seaside town of Malahide, Ireland. The scientific sessions covered a broad range of topics, including: 1) HIV pathogenesis and control, 2) reservoirs and viral expression, 3) pediatric reservoirs, 4) innate immunity and B cell responses, 5) environmental factors affecting pathogenesis, 6) loss of virologic control, and 7) HIV-2. The following article provides a brief summary of the meeting proceedings and includes a supplementary document with the meeting abstracts.

## INTRODUCTION

**Guido Silvestri** of Emory University opened the meeting with the Keynote. He gave a historical perspective of pioneering work that uncovered CD8 T cells can have a suppressive role on HIV transcription in CD4+ cells in addition to their well-described killing activity. One of the first clues that CD8s were suppressing viremia came from CD8 depletion studies, which showed reactivation of virus production in simian immune deficiency (SIV)-infected, antiretroviral therapy (ART)-treated rhesus macaques. Importantly CD4+ T cell depletion did not result in viremia suggesting this CD8 effect was not simply due to cellular proliferation following lymphodepletion. This interpretation was bolstered by evidence that latency reversal by agents such as N803 and AZD5582 is more frequently detected when CD8s are depleted in rhesus macaque studies. Overall, these data suggest that CD8 cell-mediated suppression could play a role in latency establishment and maintenance, which they are studying using an *in vitro* model. A long-term objective is to block CD8 T cell-mediated suppression of HIV transcription while stimulating CTL clearance [1–4].

## HIV PATHOGENESIS AND CONTROL

The first two sessions of the meeting focused on **HIV Pathogenesis and Control** and covered a wide range of approaches, including monitoring of Post-Treatment Controllers over time, probing mechanisms of CD4+ T cell decline, and identifying correlates of immune control.

**Stephen Migueles**, National Institutes of Health, discussed lessons learned from studying natural control of HIV that could be applied to vaccine design. He provided experimental evidence from *in vitro* analyses using HIV-infected targets that CD8+ T-cell cytotoxic capacity was significantly lower in Ad5/HIV vaccine recipients than in spontaneous HIV controllers. Low cytotoxic capacity occurred despite high-level expression of cytotoxic proteins and was attributable to impaired degranulation in response to the low antigen levels present on HIV-infected targets. This low functional avidity was due to a vaccine-induced T-cell receptor (TCR) repertoire that was polyclonal and mostly comprised of low-sensitivity TCRs. He concluded that future vaccine strategies should include measurements of functions more closely correlated with antiviral CD8+ T-cell efficacy and employ vaccines with a persisting vector and/or low MHC ligand copy number on the cell surface to select for high-avidity responses.

**Laurent Hocquelox**, Chu d'Orléans, provided an update on his longstanding cohort of Post-Treatment Controllers. He discussed the first 30 cases that were enrolled in the VISCONTI cohort, which have now been followed for 20 years. He proposed that the reservoir in post-treatment controllers resides in T cells with shorter half-lives. Intriguingly, these individuals lack strong CD8+ T cells and are associated with HLA-B35. While HLA-B35 is associated with rapid progression, it is also associated with KIR education of NK cells suggesting that NK cell activity can compensate for a weak CD8+ T cell response.

**Bonnie Howell**, Merck, presented approaches that her team at Merck are taking to reduce the reservoir. A major focus of her team is to exploit Targeted Activators of Cell Kill (TACK). These molecules are designed to induce cell death of infected cells. Currently, the team are keen to exploit the pathway where non-nucleoside reverse transcriptase inhibitors (NNRTIs) efavirenz and rilpivirine can prematurely activate intracellular protease and cleave the Caspase Recruitment Domaine-Containing Protein 8 (CARD8) inflammasome leading to cell death. A compound screen for TACK activity revealed a pyrimidone compound (Pyr01) with a strong ability to kill HIV infected cells without decreasing overall cell viability. She also covered the use of dipeptidyl peptidase 9 (DPP9) inhibitors to activate CARD8 and shared that *in vitro* assays have shown efavirenz works synergistically with DPP9 inhibitors to kill infected cells.

**Liang Shan**, Washington University, elaborated on his discovery that CARD8 inflammasome activation plays a central role in HIV pathogenesis. This work may explain the longstanding paradox: there is a massive loss of CD4+ T cells in the setting of rare infected T cells. The CARD8 inflammasome is activated immediately after HIV fusion and occurs even when reverse transcription is inhibited. Activation of this pathway may underlie accelerated CD4+ T cell loss. Mechanistically, CARD8 is activated immediately after viral fusion by viral protease leading to rapid pyroptosis. Moreover, loss-of-function mutations in the gene encoding CARD8 are found in Sooty mangabeys, which may explain why these infections are nonpathogenic in these natural hosts.

**Mirko Paiardini**, Emory University, described a new SIV-specific lymphoid tissue CD8+ T cell subset, expressing both TCF-1 and CD39, that exhibits effector and stem-like profiles. He showed the frequency of this population in rhesus macaques increases after SIV infection. While this population phenotypically carries exhaustion markers and expresses low levels of granzyme B, it degranulates and produces interferon-g when exposed to SIV peptides. The frequency of these cells correlates inversely with viral load in viremic animals and reservoir size in ART-treated rhesus macaques.

## RESERVOIRS AND VIRAL EXPRESSION

The third scientific session on **Reservoirs and Viral Expression** highlighted emerging evidence that the reservoir is neither silent nor stable.

**Una O'Doherty**, University of Pennsylvania, presented evidence from her group that the level of naïve T cell infection predicts the size and diversity of the HIV reservoir. Comparing a group of elite controllers and chronic progressors, she found elite controllers were unique for the low level of naïve T cell infection and the high levels of clonal sequences. By analyzing proviral sequences over time in elite controllers and chronic progressors on ART, she provided evidence that proviral turnover appears slower in elite controllers. Overall, individuals with higher levels of naïve T cell infection have higher proviral turnover. This result initially appears to be counterintuitive since naïve T cells have a longer intermitotic half-life. A model invoking reservoir expression by memory T cells was proposed to resolve this conundrum: infected naïve T cells differentiate into memory T cells that express HIV and therefore should turnover more rapidly than their uninfected memory counterparts. This model is consistent with continual selection over time [5].

**Mathias Lichterfeld**, Harvard University, reviewed a body of work from his group that the reservoir is often transcriptionally active, which provided additional evidence of a highly active and dynamic reservoir. Using sophisticated molecular techniques, his team characterizes proviruses over time on ART. His major finding is that proviruses detected after years of ART are often localized in gene desserts and are less transcriptionally active. This selection is more apparent in EC, but it is also apparent in some individuals who have been on ART for more than 2 decades.

Expanding on the prior talks, **Alexander Pasternak**, Amsterdam UMC, provided complementary evidence that the reservoir is more actively expressed than previously appreciated. He compared HIV cell-associated RNA expression of HIV-infected cells in 3 cohorts: elite controllers off ART, ART-naïve, and individuals on ART. Unique to this study, Dr. Pasternak carefully determined the efficiency for every step of the process of measuring HIV RNA. Using limiting dilution RT-PCR, he then quantified the frequency of infected cells expressing HIV RNA and a per-cell HIV expression level. With this approach, he revealed a remarkably high frequency of HIV RNA-expressing cells among total infected cells in ART-treated individuals.

**Alberto Bosque**, George Washington University, discussed work utilizing his recently developed TRACER assay for TRAnslational CompEtent Reservoir assay. This technology is based on an ultrasensitive planar array p24 Gag ELISA. Using this technology, the Bosque team identified a strong negative correlation between CD8+ T cell count and IL-15-induced reactivation and a positive correlation between CD4/CD8 ratio and IL-15-induced reactivation in ART suppressed patients (n=12). Overall, the TRACER assay looks to be a promising tool for reservoir assay.

The session culminated with an animated talk by **Daniel Reeves** from the Fred Hutchinson Research Center. Specifically, Dr. Reeves presented an exciting model to capture the contribution of CD4+ T cell subsets to HIV reservoir persistence using a beautiful animation. His model revealed a near equilibrium state of differentiation and death with T_CM_, T_TM_, T_EM_ contributing 10-100-fold more DNA to the reservoir than T_N_ or T_SCM_. The best models for predicting HIV-integrated DNA levels were based on cellular proliferation to a greater extent than differentiation. Taken together, this work suggests that inhibiting cellular proliferation and cellular differentiation may lead to significant reductions in reservoir size. His paper on the topic was recently published in *Nature Communications* [6].

## HIV RESERVOIRS AND LATENCY

The oral abstract session on **HIV Reservoirs and Latency** focused on new cells that contribute to the reservoir, the role of BRD4 and long non-coding RNA (lncRNA) CYTOR in perturbing HIV expression.

Later that evening **Riddhima Banga**, Lausanne University Hospital, presented evidence that the reservoir extends to lymph node dendritic cells (DCs). These DCs contain intact replication-competent HIV. Her data suggest this was not due to DC-T cell conjugates since T cell receptor excision circles (TRECS) were not detected. This work was recently published in *Cell Reports* [7].

**Ran Taube**, Ben Gurion University, reported on CYTOR, a lncRNA that activates HIV expression by binding to the HIV promoter and associating with positive transcription elongation factor B (PTEFb). In Jurkat T cells, activation profoundly increases CYTOR expression while depletion of CYTOR induced global changes in cellular RNA expression including actin pathways.

Two drugs that perturb the reservoir were discussed by **Eline Pellaers** from Zeger Debyser's group in KU Leuven. She compared the effects of 2 drugs that perturb Bromodomain-containing protein 4 (BRD4, JQ1 versus ZL0580). ZL580 increased the localization of BRD4 with acetylated histones and was associated with decreased transcription. It has additive effects with Lens epithelium-derived growth factor inhibitors (LEDGINs) suggesting there may be potential to target this pathway for block and lock approaches.

## PEDIATRIC INFECTION

The second day started with a focus on **Pediatric Infection**. All speakers emphasized the need to extend reservoir studies to children who are not “little adults.”

**Philip Goulder**, Oxford University, reported an exciting observation in South African mother-child pairs, observing that male fetuses are less susceptible to *in utero* transmission than females. They found that the virus transmitted to male fetuses was more IFN-I sensitive and of higher viral replication capacity (VRC) than those transmitted to females. In this context, he proposed that some very early cART-treated children — especially males — infected by significantly low replication capacity viruses could achieve post-treatment control without additional interventions. Overall, he suggested that early-life innate immune sex differences modulate vertical transmission, which may be critical to future interventions designed to optimize cure potential in children born with HIV.

**Ann Chahroudi**, Emory University, made the case for studying reservoirs that form during the neonatal period given the important differences between the adult and infant immune system. She emphasized the dominance of naïve T cell infection, especially in children who acquire HIV perinatally as a critical distinction. Her team is using Positron Emission Tomography (PET) scans to track SIV reservoir expression in rhesus macaques to identify tissue reservoirs and anatomic sites of early virus rebound after cessation of ART. She highlighted several examples where outcomes in pediatric studies differ from those in adults, reinforcing the importance of studying reservoirs after perinatal infection.

The role of inhibitory receptors was explored in children infected with HIV by **Hugo Soudeyns**, Centre de recherche du CHU Sainte-Justine. While inhibitory receptors are known to correlate with reservoir size in adults, this has not been studied in vertical transmission cases. He described a unique cohort of individuals infected vertically who were enrolled in Canada, termed EPIC4 (Early Pediatric Initiation-Canada Child Cure Cohort). Markers of exhaustion on CD4+ and CD8+ T cells were found to correlate with reservoir size. This correlation was particularly strong for exhaustion markers, especially PD1, on CD8+ T cells.

## INNATE IMMUNITY AND B CELL RESPONSES

The fifth session took a break from T cell biology to explore the impact of **Innate Immunity and B cell responses** on the HIV reservoir.

**Paula Cannon**, University of Southern California, provided an overview of her work on engineering B cells to express custom antibodies based on heavy chain-only antibodies (HCAb) found in camelids. She emphasized the flexibility of the platform, including the ability to accommodate non-antibody molecules and modify the Fc region. She welcomes collaborators with new ideas.

Compelling evidence that NK cells play a role in reservoir size was presented by **Maria Buzon** from Vall d'Hebron Research Institute. She first showed that a designer antibody capable of binding envelope activates NK cells and enhances antibody-dependent cell-mediated cytotoxicity (ADCC) killing of infected cells after latency reversal. This antibody consists of an Fc portion capable of binding Fc receptors on NK cells and a binding domain capable of binding the HIV envelope. However, in her mouse model, the treatment failed; yet it provided new insights. Specifically, she demonstrated this antibody expanded memory like NK cells, but reduced NK cells overall, resulting in larger reservoirs. Thus, the experiments provided a proof of concept that NK cells likely have a role in reservoir size and represent an important target.

## NEW APPROACHES TO STUDYING HIV RESERVOIRS

The oral abstract session **New Approaches to Studying HIV Reservoirs** included a barcoded virus and imaging strategies to study nuclear import.

**Alfonso Oceguera**, University of Pennsylvania, shared exciting evidence that HIV-infected cells die and divide faster than uninfected cells. Infected resting CD4+ T cells were infected with a barcoded virus and monitored for the frequency of clonal proviruses over time while simultaneously tracking cell division by eFluor. Using 3 complementary approaches, he showed that the division rate of the uninfected cells was much slower than that of the infected cells.

The role of several cellular proteins in nuclear import was explored by **Wout Hannes**, KU Leuven, from Zeger Debyser's group. These factors include Transportin SR2, Transportin 1 and Cleavage, and polyadenylation specificity factor 6. After depleting these proteins in cell lines, he infected cells with fluorescent virions prior to imaging. He thereby demonstrated there was a delay in nuclear import. Moreover, there was a change in integration sites selection as demonstrated by sequencing. He concluded that these 3 proteins have a role in nuclear import and integration site selection by HIV.

## ENVIRONMENTAL FACTORS OF PATHOGENESIS AND CONTROL

Session 6, **Environmental Factors of Pathogenesis and Control**, covered correlates of HIV rebound, including glycans (**Mohamed Abdel Mohsen**), cell-free DNA as a marker of immune control (**Satish Pillai**), and the role of inflammation in pathogenesis.

**Jean-Pierre Routy**, McGill University Health Centre, discussed evidence that acyl-CoA-binding protein is a checkpoint inhibitor for autophagy and may provide a new target for increasing autophagy. His team found elevated acyl-CoA-binding protein in people living with HIV, treated with ART is associated with inflammation and T cell dysfunction. Given that activated autophagy and low glycolysis is associated with strong CD8+ T cell responses, this could lead to new therapies to increase these responses.

**Priscilla Hsue**, University of California San Francisco, builds the case that chronic inflammation is a major contributor to cardiovascular disease in chronic viral infection. This hypothesis is supported by the correlation between the level of HIV transcription and reservoir size with atherosclerosis [8]. Moreover, elevated levels of IL-6 also play a role as they are associated with adverse cardiac outcomes in individuals with HIV or long-COVID syndrome. The increased risk of cardiovascular disease has historically been attributed to toxicity of antiretrovirals; however, there is evidence for HIV effects including elevated levels of IL-6 and a thicker intima-media of the carotid arteries. There are also parallels to individuals with long-COVID since elevated IL-6 is associated with reduced exercise capacity and chronotropic incompetence. Thus, anti-inflammatory therapies should be investigated to treat chronic inflammation in the setting of viral illness [8].

The day ended with another captivating math lecture by **Ryan Zurakowski** from the University of Delaware on lymph node reservoir dynamics. He presented the virtual lymph node model that his team developed to study antiretroviral kinetics. Using fine and coarse meshes, the team modeled drug concentrations throughout the lymph node. His team then tested the utility of their model using *ex vivo* perfused human lymph nodes.

## IMMUNE CONTROL AND LOSS OF CONTROL IN HIV-1 AND HIV-2

The last day started with a series of lectures on **Immune Control and Loss of Control in HIV-1 and HIV-2**.

**Ana Espada de Sousa**, Instituto de Medicina Molecular, captured the attention of her audience by sharing her enthusiasm for the study of HIV-2 infection. She compared the pathogenesis of HIV-2 to HIV-1, pointing out the challenges of treating HIV-2-infected individuals with undetectable or near-undetectable HIV-2. Lisbon, Portugal is a center for HIV-2 research due to its history of immigration from West Africa in the late 1990s. There are similarities between individuals infected with HIV-2 and elite controllers as they have better virologic control and very low rates of disease progression. She presented work that the GI tract mucosa is maintained in HIV-2 compared to that in HIV-1 but that there is disruption of lymph node architecture. She emphasized the distinct role of Vpx, which is preserved in HIV-2 only, resulting in lower levels of SAMHD1 in HIV-2-infected cells. Notably, HIV-2-infected individuals have stronger CD8+ T cell responses possibly related to IFN-a stimulation of the cyclic GMP-AMP synthase stimulator of interferon gene (cGAS STING) signal transduction, which is supported by data from Victor Appay (below).

Using an *in vitro* model, **Victor Appay**, University of Bordeaux, probed induction of antigen-specific responses to HIV-1 vs HIV-2. HIV-2 produced antiviral CD8+ T cells more effectively than HIV-1. Overall, T cell responses were dependent on type I interferon, which activated the cGMP-AMP synthase cGAS/STING pathway.

Additional evidence that actin pathways play important roles in HIV was illuminated by **Robert Furler-O'Brien**, Weill Cornell Medicine, using time lapse microscopy. Among HIV-infected cells, he showed 2 morphologies that resemble cytoskeletal disease involving ARP2/3. Intriguingly, these pathways are partially Nef dependent and less apparent in a subset of elite controllers.

## THE ROLE OF ART, EARLY ART AND NEW DRUGS IN HIV THERAPY

The conference ended with a session on **The Role of ART, Early ART and New Drugs in HIV therapy**. This session discussed the role of ART in elite controllers, the ability of early ART to preserve naïve T cell phenotype and function, and the potential for tyrosine kinase inhibitor intended to treat chronic myeloid leukemia (CML) to reduce the HIV reservoir.

The difficult decision “to treat or not to treat” elite controllers was explored by **Nicolas Noel** from Paris-Saclay University. He discussed the heterogeneous spectrum of controllers, spanning those with complete viral control and preserved immune systems to those who show evidence of losing control. Overall, those individuals with evidence of losing control will clearly benefit from antiviral therapy; however, the need for antiretroviral therapy in those with undetectable viral loads and small reservoirs is less clear.

The effect of early antiretrovirals on the naïve compartment was studied by **Robert Badura** at the Universidade de Lisboa. When individuals were treated very early after infection their naïve T cells were indistinguishable from those of healthy controls. Specifically, in typical HIV-1 and HIV-2 infection, CXCR3 naïve and stem cell memory are expanded. This expansion is not detected in individuals who are treated early after infection. It was also noted that HIV-2-infected individuals have an expanded CXCR3 regulatory naïve population with elevated CD95, CD23, and FOXP3 expression.

**Mayte Coiras**, Instituto de Salue Carlos III, reviewed a clinical case of a patient with HIV who was diagnosed with CML and treated with the tyrosine kinase inhibitor dasatinib. The patient experienced a remarkable 2 log reduction in HIV DNA. Moreover, lower HIV DNA persisted after dasatinib discontinuation for ~3 months at which point the patient had CML relapse and was restarted on dasatinib. Clinical trials are planned to evaluate more individuals with HIV on dasatinib therapy.

All summarized presentations and abstracts included in this article were done so with the express permission of the authors.

## SUPPLEMENTARY DATA

Supplementary materials are available at the *Pathogens and Immunity* website. Supplementary data may be provided by the authors to benefit the reader. Supplementary data are not copyedited and are the sole responsibility of the authors. Questions or comments related to supplementary materials should be addressed to the corresponding author.

FASEB 2023 Abstracts
