# Supplementary material for: Highlights from the Inaugural HIV Reservoirs and Immune Control Conference, October 1st–4th 2023, Malahide Ireland
Source: Pathog Immun. 2023 Dec 19;8(1):161–9. doi: 10.20411/pai.v8i1.653 (PMC10753932; doi:10.20411/pai.v8i1.653)
Supplement: FASEB 2023 Abstracts [file pai-8-161-s01.pdf]

FASEB Science Research CONFERENCES

Understanding Natural Immune Control of HIV-1: Implications for Vaccine Design

Stephen A. Migueles[1,2]
[1] Laboratory of Immunoregulation, NIAID, NIH, Bethesda, MD
[2] Axle Informatics, North Bethesda, MD

Background: Designing a highly effective HIV/AIDS vaccine to provide long-term protective immunity remains a major global health priority. Robust correlates of spontaneous immune control have been identified in chronic HIV-1 infection. There are reasons to be optimistic that vaccine-induced CD8+ T cells could play an important role in containing HIV below detectable levels. Herein, the functions of HIV-specific CD8+ T cells induced by current or prior candidate HIV vaccines in humans were investigated in detail and compared with chronically HIV-infected long-term nonprogressors/elite controllers (LTNP/EC) and progressors.
Aim: To investigate the mechanism underlying the low antiviral efficacy of vaccine-induced HIV-specific CD8+ T cells
Methods: PBMCs from recipients of replication defective adenovirus serotype 5 (Ad5)/HIV (Step, HVTN 071, VRC_006 and VRC_008 trials) or live, attenuated VSV-Gag (HVTN 087) vaccines were stimulated for 6 days with HIV-1-infected autologous targets or HIV-1 peptide pools and underwent negative CD8+ T-cell selection prior to restimulation with additional infected targets (cytotoxicity assessment) or PBMC targets pulsed with 10-fold serial dilutions of optimal epitopes (avidity assessment). HLA class I/HIV tetramer-labeled effectors were analyzed by flow cytometry for perforin (PRF), granzyme B (GrB) and CD107a expression. TCR sequencing was performed by tetramer+ single-cell PCR. Clonotypic diversity was assessed by calculating Simpson's Diversity Index (SDI). CD107a expression of single TCR-transduced, tetramer+ PBMCs was measured in response to HIV-infected heterologous targets matched at the restricting class I protein.
Results: CD8+ T-cell cytotoxic capacity was significantly lower in Step vaccinees than in LTNP/EC and progressors ($p<0.001$). Set point viral load post-infection in Step vaccinees was significantly, inversely correlated with CD107a+ CD8+ T-cell frequencies ($r=-0.5$, $p=0.003$). Poor killing in Ad5/HIV vaccine recipients was not a consequence of reduced cytotoxic protein expression or function. Ad5/HIV vaccinee tetramer+ cells had lower CD107a expression than LTNP/EC ($p=0.001$) and reduced loss of intracellular GrB ($p<0.001$) after restimulation with HIV-infected targets. In peptide titration experiments, CD107a expression in tetramer+ cells was significantly lower across the range of peptide dilutions in Ad5/HIV vaccinees v. LTNP/EC ($p<0.001$). These differences were most pronounced at lower concentrations (median difference 43.4% at 10e-8 M, $p<0.001$), consistent with lower functional avidity. Median interpolated peptide concentration on the surface of infected targets was 10e-8 M. The TCR repertoire of HIV-specific cells was more diverse in vaccinees than in LTNP/EC (0.83 v. 0.51 SDI, $p=0.02$). In response to infected targets, vaccinee TCR transductants exhibited reduced degranulation compared with to LTNP/EC transductants ($p=0.01$), indicating that reduced functionality was mediated at the level of the antigen receptor.
Conclusion: Several candidate HIV vaccines failed to select for CD8+ T cells of sufficient avidity to mediate high-level degranulation and cytotoxicity in response to the low peptide-MHC levels on the surface of HIV-infected targets. These results define a mechanism accounting for poor cytotoxic capacity and antiviral activity induced by these vaccines and suggest that an effective CD8+ T-cell responses may require a vaccination strategy that drives further TCR clonal selection.

 O'Doherty U, Picado JM, Cirion AS. Highlights from the Inaugural HIV Reservoirs and Immune Control Conference, October 1st- 4th 2023, Malahide Ireland. *Pathogens and Immunity*. 2023;8(1):161-169.doi: 10.20411/pai.v8i1.653

Post-Treatment Controllers

Laurent HOCQUELOUX [1]
[1] Orléans University Hospital, Orléans, France

History: In the early 2000s, structured treatment interruptions were offered to many people with HIV (PWH), with the objective to induce "self-vaccination". The first clustered cases of PWH able to maintain sustained undetectability of HIV despite complete cessation of ART were observed in this context. Later, similar cases were replicated in clinical trials evaluating ART initiated at the time of primary HIV infection (PHI). Two cohorts, one French and one American, have set out to describe this phenomenon of post-therapeutic control and elucidate its mechanisms.

Definition: There is no consensual definition of Post-Treatment Controllers (PTCs). In the French prospective VISCONTI cohort, PWH included meet three consecutive conditions. Firstly, PWH (adults or children) must not have spontaneous HIV control: the viral load measured before ART must be greater than 2000 copies/mL (this criterion can be omitted in the case of infected children treated from birth). Secondly, PWH must have received ART for at least twelve months, under which they became undetectable. Thirdly, they must have maintained an undetectable viral load (or less than 400 copies/mL) for at least twelve months, after ART have been completely stopped.

Frequency: PTCs are even rarer than natural HIV controllers (HICs), and have been described mainly in the context of early-ART (i.e., since the PHI), where 5-15% of PWH could achieve this PTC status. Although controversial, data suggest the existence of PTCs treated during the chronic phase of HIV infection, but much less frequently than during PHI.

Characteristics and long-term follow-up: In the VISCONTI cohort, analysis of the first 30 cases included (29 adults, 1 child) revealed mainly Caucasian men (2/3 of them). Half the cases were men who had sex with men. Most PTCs had received ART at the time of PHI, which was symptomatic in ¾ of cases (median viral load = 5.2 Log copies/mL). At the time of discontinuation, PTCs were a median of 38 years old, had received ART for a median of 3.8 years, and had restored a near-normal CD4 count and CD4/CD8 ratio >1. The median duration of treatment-free follow-up was 13 years (up to 20 years), during which no PTC had an AIDS event. During this follow-up, 16/30 PTCs (53%) had optimal virological control (99% of viral loads below threshold), the other 14/30 (47%) having intermittent replication. Overall, PTCs shared several remarkable features: a very low level of total HIV DNA in PBMCs (median = 1.85 Log copies/10^6 PBMCs), a high stability of absolute CD4 counts and CD4/CD8 ratio over time, and finally a low level of lymphocyte activation and pro-inflammatory cytokines (lower than the majority of HICs and close to healthy donors). At the end of follow-up, 24/30 PTCs (80%) remained ART-free, while 6/30 (20%) resumed ART mostly because of virological failure (i.e., 2 consecutive plasma viral load >400 copies/mL).

In the American CHAMP cohort, where selection of PTCs at entry was less strict than in VISCONTI, about 1/5 of them remained undetectable at 5 years.

Mechanisms of virological control: PTCs carry a virus capable of replicating and reinfecting other lymphocytes. However, early-ART appears to have greatly reduced the reservoir, viral diversity, the number of intact integrated sequences and forced the virus to persist in quiescent short half-life CD4+ cells (mainly memory transitional and memory effector cells). This could explain the gradual decline in the reservoir observed in some PTCs after discontinuation of ART. Unlike HICs, PTCs do not have a strong cytotoxic CD8-specific lymphocyte response, although their CD4- and CD8-specific responses can be polyfunctional.

Remarkably and paradoxically, PTCs are not enriched in HLA B*27 and/or B*57, but rather in B*35, which is associated with a more severe PHI and more rapid progression to AIDS, in the absence of ART. A particular MHC-genotype (Bw4TTC2), congruent with KIR-education of NK cells, is present in all HLA-B*35 PTCs from the VISCONTI study and is associated with higher probability to durably maintain undetectable viremia after ART interruption in early-treated PWH carrying HLA-B*35 alleles. These results support a preponderant role of NK cells in post-treatment control, ensuring a strong NK cell response, capable of killing infected cells. Finally, data suggest that early-ART may have preserved the integrity of gut anatomical and functional lymphoid structures, which may have benefited the preservation of an effective specific response and less residual immune activation.
Conclusion and perspectives: Understanding PTCs (where genetics, immune responses and early therapeutic intervention lead to sustained virological control) can help us design a cure for HIV or, at least, better identify PWH likely to benefit from drastic therapeutic alleviation.

CD8+ Lymphocytes Modulate Stemness and Survival Pathways in HIV-Infected CD4+ T Cells to Promote HIV Latency

Deanna A. Kulpa[1]
[1]Department of Pathology and Laboratory Medicine, Emory University School of Medicine

The persistence of HIV infection during ART is due to a reservoir of latently infected cells that harbor replication-competent virus and evade immune recognition. Defining the mechanisms responsible for the establishment and maintenance of HIV latency is crucial to target and eliminate HIV-infected cells. Previous studies demonstrated that CD8+ T-cells inhibit virus replication during untreated HIV/SIV infection and inhibit virus production during ART; however, the mechanisms responsible for this antiviral effect remain poorly understood.

We used a primary cell-based *in vitro* HIV latency model to examine non-cytotoxic suppression of HIV transcription by CD8+ T cells. Memory CD4+ T-cells from HIV-naïve individuals were infected *in vitro* and then co-cultured with activated autologous CD8+ T-cells in the presence of ART. To evaluate CD8+ T-cell suppression activity, we quantified HIV-gag expression by flow cytometry and integrated HIV DNA frequency by qPCR. We then employed a combination of high-dimensional and transcriptomic analyses to identify CD4+ T-cell signaling pathways differentially modulated by the co-culture with CD8+ T-cells associated with the reduction in HIV expression.

We observed an average of 50% reduction in HIV expression from memory CD4+ T-cells when co-cultured with autologous activated CD8+ T-cells at 1:1 ratio. This viral suppression occurred in the absence of elimination of the HIV-infected cells. Further experiments demonstrated the suppression activity of CD8 T cells was mediated through a non-MHC-I mechanism. Transcriptional analyses showed that co-cultured CD4+ T-cells adopted a resting phenotype, with downregulation of cell cycle, pro-inflammatory and apoptosis signaling. Pathways previously associated with HIV production were also downregulated, including oxidative phosphorylation and the mechanistic target of rapamycin complex 1. Of note, co-culture with CD8+ T-cells activated Wnt/β-catenin signaling and stem-cell memory pathways in HIV-infected memory CD4+ T-cells.

Our studies demonstrate that co-culture with CD8+ T-cells promotes changes in metabolic and cell survival pathways in HIV-infected memory CD4+ T-cells that may negatively regulate HIV expression and ultimately promote the persistence of latency. Modulation of this CD8-mediated activity may represent a tool to disrupt HIV latency and reservoir persistence in ART-treated individuals.

This work was supported by UM1AI164562, co-funded by National Heart, Lung and Blood Institute, National Institute of Diabetes and Digestive and Kidney Diseases, National Institute of Neurological Disorders and Stroke, National Institute on Mental Health, National Institute on Drug Abuse and the National Institute of Allergy and Infectious Diseases and R01AI143414 funded by the National Institute of Allergy and Infectious Diseases, United States.

Exploring the role of Naïve T Cells in reservoir turnover

Sam Weissman[1], Alfonso Oceguera[1], Ashley M Ginda[1], Marilia Pinzone[1], Liam Swiggard[1], LaMont Cannon[1,2], Ryan Zurakowski[3], Stephen Migueles[4], Una O'Doherty[1]
[1] Department of Pathology and Laboratory Medicine, University of Pennsylvania, Philadelphia PA
[2] Center for the Study of Biological Complexity, Virginia Commonwealth University, VA
[3] Department of Biomedical Engineering, University of Delaware, Newark, DE
[4] Laboratory of Immunoregulation, NIAID, NIH, Bethesda, MD

Introduction: The role of naïve T cells in HIV persistence and reservoir dynamics is underexplored. Naive T cells have a distinct reservoir composition compared to other T cell subsets with higher diversity and lower clonality. The unique properties of Naïve T cells make them an ideal reservoir including their longevity, resistance to immune clearance, resistance to reactivation and their fundamental role in memory repopulation. We previously studied these properties of naïve infection in a cohort of elite controllers and chronic progressors selected to represent a range of reservoir sizes and immune control. Proviral sequencing of CD4 T cells subsets revealed that naïve T cell infection were strong predictors of HIV reservoir size and diversity. In the current study we return to our prior cohort to assess the role of naïve infection on HIV reservoir turnover. We compare and contrast elite controllers with low levels of naïve infection to chronic progressors with high levels of naïve infection.
Method: Clonal overlap of multiple paired time points was determined using longitudinal proviral sequences from a cohort of elite controllers and chronic progressors. Simultaneously, T cell receptor sequencing was performed to provide a baseline measure of T cell turnover in the same samples. To visualize clonal overlap, Venn Diagrams were generated from proviral and TCR sequences and a Morisita distance was calculated to provide a standardized metric of clonal overlap.
Results: While proviral clonal overlap diminished over time in both cohorts, overlap contracts more rapidly in chronic progressors than in elite controllers. Our analysis also suggests that infected T cells turnover faster than uninfected T cells.
Conclusions: Turnover of infected T cells appears to be slower in elite controllers compared to chronic progressors. We speculate the lower levels of naïve infection found in elite controllers leads to diminished repopulation of memory subsets which leads to slower turnover.

Chromosomal Integration sites as biomarkers of HIV-1 reservoir cell selection

Mathias Lichterfeld[1]
[1]Brigham and Women's Hospital and Ragon Institute of MGH, MIT, and Harvard

With more than 38 million people living with HIV-1 worldwide, developing a cure for HIV-1 remains a major global health priority. Lifelong persistence of HIV-1 is frequently attributed to a pool of stable, transcriptionally silent HIV-1 proviruses, which are unaffected by currently available antiretroviral therapy (ART) or host immune activity. Our recent work suggests a more dynamic evolution of the viral reservoir, primarily characterized by a longitudinal selection of genome-intact proviruses integrated in heterochromatin locations.

These proviruses are frequently transcriptionally repressed, which likely protects them from host immune recognition and seems to confer a longitudinal selection advantage. Such selection mechanisms appear to be most obvious in persons with natural (drug-free) control of HIV-1, but are also detectable in individuals undergoing suppressive antiretroviral therapy for very long periods of time (>20 years). While the immunological mechanisms underlying this selection process are not clear at this point, recent data from our lab demonstrate that selection of proviruses can be accelerated through the pharmacological administration of latency reversing agents; for instance, we noted in a randomized controlled human clinical trial that during treatment with the histone deacetylase inhibitor Panobinostat, proviruses in proximity to H3K27ac marks were preferentially eliminated, while proviruses in greater distance to acetylated histones persisted. Selection of proviruses in deeper latency over time may act in favor of the host and could be regarded as a therapeutic objective for clinical studies aiming for HIV-1 cure. Moreover, longitudinal monitoring of HIV-1 integration sites may allow to unravel immune selection processed that remain masked when viral reservoir analysis is solely based on quantitative evaluations of intact proviral frequencies.

CD4/CD8 ratio positively correlates with reactivation of translation-competent virus in peripheral CD4 T cells *ex vivo*

J. Natalie Howard[1], Callie Levinger[1], Rémi Fromentin[2], Nicolas Chomont[2], and Alberto Bosque[1]
[1]Department of Microbiology, Immunology, and Tropical Medicine, George Washington University, Washington, DC
[2] Université de Montréal, Centre de recherche du CHUM, Montréal, QC, Canada

Background: The latent HIV reservoir remains the largest barrier to cure despite effective antiretroviral therapy (ART). Most assays measuring viral reactivation rely on stimulation of cells *ex vivo* with latency reversing agents (LRA) and quantification of viral RNA. In this work we aimed to evaluate the ability of IL-15 and αCD3/αCD28 to reactivate the translation-competent viral reservoir in CD4 T cells isolated from ART-suppressed people with HIV (PWH).
Methods: We optimized a novel assay to evaluate TRAnslational-CompEtent viral Reservoirs (TRACER Assay) using a planar array ultrasensitive p24 Gag ELISA. We have previously shown that this assay can detect viral p24 at fg/ml in both supernatants and cell lysates (Levinger et al., *Scientific Reports*, 2021). Using this assay, we evaluated the ability of IL-15 and αCD3/αCD28 to reactivate the translation-competent viral

reservoir in CD4 T cells from 12 ART-suppressed participants and correlated viral reactivation with different markers of HIV persistence and other clinical characteristics.
Results: IL-15 and αCD3/αCD28 both reactivated translationally competent virus in 9 out of 12 participants. In responders, viral reactivation by IL-15 when compared to unstimulated was an average of 14-fold (3.4-37.3) in supernatants ($p = 0.0039$) and an average of 387-fold (1.05-724) by αCD3/αCD28 ($p = 0.0039$). Levels of p24 induced by IL-15 and αCD3/αCD28 were positively correlated ($r = 0.79$, $p = 0.048$). On average, IL-15 reactivated 43% of that of αCD3/αCD28 (median 15%, range 0.04-266%). No correlation was observed between reactivation of translationally competent virus by both stimuli and markers of persistence including total HIV DNA, integrated HIV DNA, TILDA, or HIV flow. Interestingly, we observed a strong negative correlation between absolute CD8 T cell counts with IL-15 reactivation levels ($r = -0.78$, $p = 0.0043$) and with αCD3/αCD28 stimulation to a lesser degree ($r = -0.52$, $p = 0.089$). We also observed a strong positive correlation between the CD4/CD8 ratio and IL-15 reactivation ($r = 0.72$, $p = 0.011$), while no correlation was observed between CD4/CD8 ratio and αCD3/αCD28 stimulation ($r = 0.25$, $p = 0.43$). No correlation was observed between either IL-15 or αCD3/αCD28 reactivation levels with absolute CD4 T cell counts or the proportion of CD4 T cell subsets CD45RA+, CD45RA-, naïve (CD45RA+/CD27+/CCR7+), central memory (CD45RA-/CD27+/CCR7+), transitional memory (CD45RA-/CD27+/CCR7-), effector memory (CD45RA-/CD27-/CCR7-), or terminally differentiated (CD45RA+/CD27-/CCR7-).
Conclusion: We have developed a new assay to measure reactivation of translational-competent virus *ex vivo* from ART-suppressed PWH and have identified CD4/CD8 ratio and absolute CD8 T cell levels as potential markers of translational-competent reservoirs in peripheral CD4T cells.

HIV passenger kinetics during CD4 T cell subset proliferation and differentiation

Daniel B. Reeves[*1,2], Charline Bacchus-Souffan[*3], Mark Fitch[4], Mohamed Abdel-Mohsen[5], Rebecca Hoh[6], Haelee Ahn[7], Mars Stone[8], Rick Hecht[7], Jeff Martin[9], Steven G. Deeks[6], Marc K. Hellerstein[4], Joseph M. McCune[10], Joshua T. Schiffer[**1,11,12], Peter W. Hunt[**7]
[1]Vaccine and Infectious Disease Division, Fred Hutchinson Cancer Center, Seattle, WA
[2]Department of Global Health, University of Washington, Seattle, WA
[3]Vir Biotechnology, Inc. San Francisco, CA
[4]Department of Nutritional Sciences and Toxicology, University of California, University Avenue and Oxford St, Berkeley, CA
[5]The Wistar Institute, 3601 Spruce St, Philadelphia, PA
[6]Department of Medicine, Zuckerberg San Francisco General Hospital, University of California, San Francisco, CA
[7]Division of Experimental Medicine, Department of Medicine, University of California San Francisco, San Francisco, CA
[8]Vitalant Research Institute, San Francisco, CA
[9]Epidemiology & Biostatistics, University of California San Francisco School of Medicine, San Francisco, CA
[10]HIV Frontiers, Global Health Accelerator, Bill & Melinda Gates Foundation, Seattle, WA
[11]Clinical Research Division, Fred Hutchinson Cancer Center, Seattle, WA
[12]Department of Allergy and Infectious Diseases, School of Medicine, University of Washington, Seattle, WA
*,** Equal contribution

Abstract
Persistence of HIV in people living with HIV (PWH) on suppressive antiretroviral therapy (ART) has been linked to physiological mechanisms of CD4+ T cells. Here, in the same PWH on ART we measure longitudinal kinetics of HIV DNA and T cell turnover rates in 5 CD4 subsets: naïve (T_N), stem-cell- (T_{SCM}), central- (T_{CM}), transitional- (T_{TM}), and effector-memory (T_{EM}). HIV decreases in T_{TM} and T_{EM} but not in less-differentiated subsets. Cell turnover is ~10 times faster than HIV clearance in memory subsets, implying that cellular proliferation consistently creates HIV DNA. The optimal mathematical model for these integrated data sets posits HIV DNA also passages between CD4+ T cell subsets via cellular differentiation. Estimates are heterogeneous, but in an average participant's year ~10 (in T_N and T_{SCM}) and ~10^4 (in T_{CM}, T_{TM}, T_{EM}) proviruses are generated by proliferation while ~10^3 proviruses passage via cell differentiation (per million CD4). In simulations, therapies blocking proliferation and/or enhancing differentiation could reduce HIV DNA by 1-2 logs over 3 years. In summary, HIV exploits cellular proliferation and differentiation to persist during ART but clears faster in more proliferative/differentiated CD4+ T cell subsets. The same physiological mechanisms sustaining HIV might be temporarily modified to reduce it.

Characterization of the Gastrointestinal-Resident Latent HIV-1 Reservoir with Single-Cell RNA Sequencing

Jackson J Peterson[1,2], Elizabeth S Bennett[2], Joshua Fox[2], Cory H White[3], Katherine James[2], Brigitte Allard[2], Matt Clohosey[2], Shipra Chandel[2], David M Margolis[2,1], Anne-Marie Turner[2], Nancie Archin*[2], Edward P Browne*[2,1]
[1]UNC-Chapel Hill , Microbiology and Immunology, Chapel Hill, NC
[2]UNC-Chapel Hill, HIV Cure Center, Institute of Global Health and Infectious Diseases, Chapel Hill, NC
[3]Merck, Merck Exploratory Science Center, Cambridge, MA

*These authors contributed equally to this work.

Background: Human gastrointestinal (GI) tissues are a major site of HIV-1 viral replication and persistence, but the nature of the persistent HIV-1 reservoir in the GI remains poorly described. Previous study has identified higher levels of HIV viral DNA but lower levels of HIV transcriptional initiation in the colon as compared to the peripheral blood. Recently the immunology field has described distinct transcriptional characteristics in GI tissue resident T cells which facilitate tissue trafficking, retention, and localized immune responses, but it is unclear mechanistically how biological differences in GI tissue resident T cells may contribute to HIV persistence. Here we aimed to characterize HIV infected cells in GI tissue from people with HIV (PWH) who are well suppressed on antiretroviral therapy (ART).
Methods: We developed a protocol to isolate and sequence GI-associated human lymphocytes with 10x Genomics scRNAseq. We analyzed 46,274 high-quality single-cell transcriptomes from GI-associated cells, primarily T and B cells, from three PWH virally-suppressed on ART. As a comparator we sequenced 53,118 peripheral blood mononuclear cells (PBMCs) from the same individuals; in both cases, a subset of cells were treated with PMA/ionomycin to induce viral gene expression. Since traditional scRNA seq techniques employ short-read sequencing and are limited in terms of their ability to characterize viral transcripts with respect to splicing patterns and inactivating mutations, we have also developed an approach to derive more detailed viral

sequence information from scRNAseq. We employed a novel PCR approach to amplify viral cDNAs from a cDNA pool produced in an intermediate step of the scRNA seq library protocol. Amplified viral cDNAs were sequenced with long-read Oxford Nanopore sequencing using a strategy that also sequences the cell barcodes.
Results: In total we identified 9 HIV RNA positive cells from GI samples and 6 HIV RNA positive cells from peripheral blood. The putative infected cells exclusively cluster with T cells, albeit very few macrophages were sequenced in the overall dataset. We identify expected patterns of compartment-specific gene expression in GI tissue resident T cells. Non-stimulated GI resident T cells differentially express surface factors including ITGA1/CD103 and CD69 whereas non-stimulated peripheral blood T cells express SELL/CD62L, CCR7, and S1PR1. Non-stimulated GI resident T cells express distinct transcriptional regulators like RUNX3 and STAT4 whereas non-stimulated peripheral blood T cells express KLF2, LEF1, and TCF7/TCF1. We further explore tissue-specific differences in the expression of factors known to influence HIV replication and latency.
Using an approach involving PCR amplification and long-read sequencing of viral cDNAs from scRNA seq libraries, we determined that 10x Genomics scRNA sequencing exhibits bias towards small and/or aberrantly primed viral transcripts. This limitation arises from low processivity of the reverse-transcription step employed during scRNA seq sample preparation.
Conclusion: Here we examine T cells from the GI latent reservoir and characterize important biological differences compared to more easily examined peripheral blood T cells. By identifying molecular mechanisms of differential transcriptional regulation in GI tissue resident T cells we generate testable hypotheses that may lead to a better understanding of HIV latency and persistence. Furthermore, we make important technical insights into the detection of HIV RNA by scRNA seq that will guide future work studying HIV persistence with single cell transcriptomics.

Lymph node dendritic cells harbor inducible replication competent HIV despite years of suppressive ART

Riddhima Banga[1], Francesco Andrea Procopio[1], Erica Lana[1], Annamaria Kauzlaric[3], Mathilde Foglierini[1], Oscar Alfageme Abello[1], Susanna H.M. Sluka[4], Olivia Munoz[1], Andrea Mastrangelo[1], Craig Fenwick[1], Yannick Muller[1], Matthias Cavassini[5], Rafael Trunfio[6], Sébastien Déglise[6], Mathias Lichterfeld[2,7], Giuseppe Pantaleo[1,8], Matthieu Perreau[1]
[1]Services of Immunology and Allergy, Lausanne University Hospital, University of Lausanne, 1011 Lausanne, Switzerland
[2]Ragon Institute of MGH, MIT and Harvard, Cambridge, MA
[3]Translational bioinformatics and statistics Department of Oncology, University of Lausanne Swiss Cancer Center, Lausanne, Switzerland
[4]Newborn Screening Switzerland, University Children's Hospital Zurich, University of Zurich, Zurich, Switzerland,1011 Lausanne, Switzerland
[5]Services of Infectious Diseases, Lausanne University Hospital, University of Lausanne, 1011 Lausanne, Switzerland
[6]Services of Vascular Surgery, Lausanne University Hospital, University of Lausanne, 1011 Lausanne, Switzerland
[7]Infectious Disease Division, Brigham and Women's Hospital, Boston, MA
[8]Swiss Vaccine Research Institute, Lausanne University Hospital, University of Lausanne, 1011 Lausanne, Switzerland

Background: The comprehensive characterization and quantification of the HIV tissue reservoirs is required to design appropriate therapeutic intervention(s) to achieve a cure. While multiple studies demonstrated the preponderant role of CD4 T cells in HIV persistence, the role of dendritic cells (DCs) has long time been neglected, probably because of their short lifespan, their low frequency, the scarcity of human tissues and a strong dogma established from *in vitro* derived DCs. In the present study, we wished to unravel the contribution of the two major lymph node (LN) DC subpopulations (resident *versus* migratory DCs) to HIV persistence.
Methods: To address this issue, we performed 1) an in-depth transcriptomic, phenotypic and functional characterization of LN DCs; 2) assessment of the susceptibility of LN DCs to HIV infection *in vitro*; 3) assessment of major virological parameters associated with HIV persistence in LN DCs isolated from viremic and aviremic ART treated HIV-infected subjects.
Results: We showed that the two major *i.e.* LN migratory (Lin-HLADR+CD45+CD11c+CCR7+) and resident (Lin-HLA-DR+CD45+CD11c+CCR7-) DCs were susceptible to HIV infection and supported active cycles of *de novo* viral replication *in vitro* ($P<0.05$). In addition, LN resident and migratory DCs isolated from viremic individuals contained intact HIV provirus, were transcriptionally active directly *ex vivo* and were capable of producing HIV RNA/p24 upon TLR7/8 stimulation *in vitro* ($P<0.05$). Interestingly, both LN DC subpopulations isolated from ART treated HIV-infected individuals contained HIV intact provirus and inducible replication competent HIV despite the expression of the anti-viral restriction factor SAMHD1. Of note, HIV-1 RNA was consistently detected in culture supernatants of LN migratory DCs from HIV-infected individuals who were treated for up to 14 years and detectable in culture supernatant of LN resident DCs in one individual treated for up to 19 years.
Conclusions: These findings indicate that LN DCs isolated from ART-treated aviremic HIV-infected individuals may represent a yet untapped reservoir of infectious HIV in LN tissues.

Role of Long Non-Coding RNAs (lncRNAs) in HIV Latency

Alona Kuzmina, Ran Taube
Ben-Gurion University, The Shraga Segal Microbiology Immunology and GEnetics, Beer Sheva, Israel

Anti-retroviral therapy (ART) has a profound inhibitory impact on infection by the human immunodeficiency virus (HIV). However, for most HIV carriers, interruption of ART leads to a rapid rebound in viral load, as the virus persists in a latent state within an infected reservoir that is comprised primarily of resting memory CD4 T cells. To eliminate the latent reservoir, steps that control HIV gene expression and viral latency need to be elucidated. However, while the mechanisms by which host proteins govern HIV gene expression and viral latency are relatively well-studied, the emerging role of non-coding RNAs (ncRNA) in the context of T cell activation, HIV gene expression and viral latency has not yet been carefully explored.
Here, we documente changes in the transcriptome of HIV-infected Jurkat-derived T cells in response to T cell activation. We report of a marked change in cellular gene expression, including that of protein-coding genes and ncRNAs. Among those with the most profund shift in RNA levels following T cell stimulation, we identified Cytoskeleton Regulator RNA (Cytor) lncRNA. Gain and loss-of-function studies in both CD4 T cell lines and in primary cells show that following stimulation, Cytor activates viral gene expression and suppresses latency establishment. Mechanistically, Cytor directly occupies the HIV promoter and associates with the cellular positive transcription elongation factor (P-TEFb) to activate viral gene expression. Indirectly, Cytor depletion

leads to broad changes in the expression of cellular genes including the control of actin dynamic pathways. Knockdown of Cytor expression disrupts cytoplasmic actin polymerization in response to T cell stimulation, and treatment of HIV-infected T cells with actin polymerization inhibitors inhibits HIV gene expression. We conclude that direct and indirect effects of Cytor lncRNA control of HIV gene expression and viral latency.

New Horizons for BRD4 Modulators in a Block-and-Lock Functional Cure of HIV-1 Infection

Eline Pellaers, Lore Wils, Anayat Bhat, Frauke Christ, Zeger Debyser
Molecular Virology and Gene Therapy, KU Leuven, Leuven, Belgium

Background: The persistence of HIV-1 provirus in a transcriptionally silent state in long-lived cells of the immune, known as the latent reservoir, is the main impediment for an HIV-1 cure. Using latency promoting agents (LPAs), the block-and-lock cure strategy aims to establish a cellular reservoir unable to reactivate after treatment discontinuation. However, new drug targets for the development of LPAs are crucial. The epigenetic reader, bromodomain-containing protein 4 (BRD4), plays a role in the establishment/maintenance of latency by regulating HIV-1 transcription. Most BRD4 modulators, such as JQ1, are known to reactivate latency. However, recently, the first BRD4 modulator was reported that epigenetically represses HIV-1 transcription, named ZL0580.
Aims: To gain insight into the role of BRD4 in the transcriptional regulation of HIV-1 and to validate BRD4 as a block-and-lock cure target, we now investigated the effect of JQ1 and ZL0580 on HIV-1 transcription side-by-side as well as their mechanism of action. Moreover, we assessed the efficiency of latency promoting cocktails including ZL0580 and LEDGINs (CX014442). LEDGINs are well-characterized LPAs, which inhibit the interaction between viral integrase and lens epithelium-derived growth factor (LEDGF/p75). As a result, LEDGINs reduce viral integration and retarget the residual provirus to regions resistant to reactivation.
Methods: SupT1 cells were infected with pNL4.3 HIV-virus. After ten days, cells were treated with JQ1/ZL0580, and reactivated with TNF-α (10 ng/mL). 24 h post-reactivation, samples were harvested for the luciferase reporter assay. In addition, enriched CD4+T cells were stimulated with 10 μg/mL phytohemagglutinin (PHA) two days before transduction. These cells were transduced with pNL4.3 HIV-virus and meanwhile treated with a dilution series of ZL0580. On day three and seven post-transduction, samples were collected for the luciferase reporter assay. To investigate the mechanism of action, SupT1 cells treated with JQ1/ZL0580 were fixated for single-cell imaging of both vDNA and vRNA. Moreover, co-localization between BRD4 and H3K9/14Ac in presence of JQ1/ZL0580 was examined with confocal imaging and quantified via Pearson correlation coefficient. Finally, ZL0580 was combined with LEDGINs (CX014442) in SupT1 cells. Same experiments as described above were conducted with cells treated with LEDGIN (CX014442) during infection.
Results: In the HIV luciferase reporter assay, JQ1 promoted and ZL0580 hampered HIV-1 transcription, both in cell lines and primary lymphocytes. Single-cell imaging of viral DNA and RNA of cells treated with JQ1 or ZL0580 demonstrated that both compounds work on the transcriptional level. In addition, we corroborated that JQ1 decreases and ZL0580 increases the co-localization of BRD4 with acetylated histones. Finally, we combined LEDGINs (CX014442) with BRD4 modulators. Interestingly, ZL0580 showed an additive effect in combination with LEDGINs (CX014442) in blocking transcription and locking reactivation.
Conclusion: Collectively, our results imply a multifaced role of BRD4 in HIV-1 transcription and the potential of BRD4 to be modulated by small molecules to both stimulate and hamper HIV-1 transcription. In. addition,

our results show that JQ1 and ZL0580 both work on the transcriptional level but oppositely affect the interaction of BRD4 with the acetylated histones. Finally, we showed that latency promoting cocktails including ZL0580 increase the efficiency of block-and-lock therapies.
This research was funded by the Research Foundation Flanders (FWO) (G0A5316N and SBO–Saphir) and the KU Leuven Research Council (C14/17/095–3M170311). E. P. has been funded by a fellowship of FWO.

Taming the Viral Reservoir over Three Decades of Advancements in HIV Treatment

Irene González-Navarro[1], Víctor Urrea[1], Cristina Gálvez[1], Beatriz Mothe[1,2,3,4], Lucía Bailón[2,5], Javier Martinez-Picado[1,3,4,6], Maria Salgado[1,3]
[1] IrsiCaixa AIDS Research Institute and Institute for Health Science Research Germans Trias i Pujol (IGTP), Badalona, Spain
[2] Fight Infections Foundation, Infectious Diseases Department, Hospital Universitari Germans Trias i Pujol, Badalona, Spain
[3] CIBERINFEC, Carlos III Health Institute (ISCIII), Madrid, Spain
[4] Chair in Infectious Diseases and Immunity, University of Vic – Central University of Catalonia (UVic-UCC), Vic, Spain
[5] Department of Medicine, Autonomous University of Barcelona, Catalonia, Spain
[6] Catalan Institution for Research and Advanced Studies (ICREA), Barcelona, Spain

Background: Since the advent of combination antiretroviral therapy (ART), the clinical management of HIV infection has steadily improved, not only because of the availability of more potent and safer drugs, but also because of recommendations for universal treatment at diagnosis, regardless of disease stage and CD4 count, and efforts to increase HIV diagnose at its earliest stage after HIV acquisition. However, little is known about the effects of such improvements on the establishment of the latent HIV reservoir. Here, we characterize a large group of people with HIV (PWH) on ART to determine the influence of multiple factors on the evolution of the HIV reservoir.
Methods: We analyzed the reservoir of 893 PWH treated and virologically suppressed for >3 years by measuring total HIV-DNA in PBMCs by ddPCR. Those with <50 HIV-DNA copies/10^6 PBMCs were classified as LoViReT. 40 demographic, clinical, virologic, and immunologic variables were collected to explore their association with the LoViReT status using the Random Forest machine learning algorithm, and additional methods such as LOESS and logistic regression, or PCA.
Results: 180 (20%) of the 893 PWH were classified as LoViReTs. Minimum CD4 counts and maximum viremia during clinical follow-up, as well as shorter time from ART initiation to viral suppression and longer time on an integrase strand transfer inhibitor (INSTI)-based regimen predicted LoViReT status (classification error – 30%). The multiple logistic regression model estimation of the effect of these parameters was: minimum CD4 counts OR=1.52 (per 100 cells/µL), maximum viremia OR=0.73 ($\log_{10}$ plasma HIV-1-RNA copies/mL), and time to achieve suppression OR=0.59 (years). We further analyzed how those factors fluctuate based on ART start date. We observed a decrease in total HIV-DNA and a greater percentage of LoViReTs when ART was started after 2007. Indeed, time from treatment to suppression changed from 1.7 years in 1998 to <4 months in 2020. Since INSTIs were introduced in 2007, we performed a sub-analysis of the effect of INSTI-based regimens on HIV proviral levels, noting that individuals initiating ART with INSTIs had lower reservoir levels (p=0.001) and shorter times to undetectable viremia ($p=2.2x10^{-16}$).

Conclusion: The constant improvement in ART guidelines and the introduction of new generation drugs are related to the establishment of lower levels of the HIV reservoir in PWH and, therefore, to the increase in the LoViReT phenotype among the individuals examined, which could facilitate the future success of medical strategies aimed at achieving a functional cure.
Keywords: Established reservoir, HIV treatment guidelines, ART initiation, $CD4^+$ T-cell counts, novel antiretroviral drugs
This research was funded by grant #UM1 AI164561 from Martin Delaney Collaboratories for HIV Cure Research (NIH/NIAID).

Expression of Inhibitory Receptors on CD4+ and CD8+ T Cells is Associated with the Size o the Viral Reservoir in Children, Adolescents and Young Adults with Vertically-Acquired HIV Infection

Hugo Soudeyns[1,2,3,4], Hinatea Dieumegard[1,2], Madeleine Aby Diallo[1,2], Fatima Kakkar[3,4], Ari Bitnun[5], Stanley Read[5], Jason Brophy[6], Lindy Samson[6], Michael T. Hawkes[7], EPIC[4] Study Group
[1]Unité d'immunopathologie virale, Centre de recherche du CHU Sainte-Justine, Montreal, Canada
[2]Departement of Microbiology, Infectiology & Immunology, Faculty of Medicine, Université de Montréal, Montreal, Canada
[3]Centre d'infectiologie mère-enfant, Centre de recherche du CHU Sainte-Justine, Montreal, Canada
[4]Departement of Pediatrics, Faculty of Medicine, Université de Montréal, Montreal, Canada
[5]Hospital for Sick Children, Toronto, Canada
[6]Children's Hospital of Eastern Ontario, Ottawa, Canada
[7]Department of Pediatrics, University of British Columbia, Vancouver, Canada

Background: In adults, HIV infection leads to the expression of so-called inhibitory receptors at the surface of $CD4^+$ and $CD8^+$ T lymphocytes, expression which is associated with T cell exhaustion. In addition, the HIV reservoir is largely comprised of $CD4^+$ T cells that express inhibitory receptors PD-1 and TIGIT. However, there is a paucity of analogous information on HIV-infected children and on how these parameters evolve between childhood, adolescence and young adulthood.
Aims: The objective of this study was to characterize the patterns of expression of inhibitory receptors and their association with the size of HIV reservoir in children, adolescents and young adults with vertically-acquired HIV infection.
Methods: Multi-parameter flow cytometry was used to assess expression of cell-surface markers associated with lineage (CD3, CD4, CD8, CD45RA, CCR7), activation (CD69, HLA-DR) and T cell exhaustion (PD-1, TIM-3, LAG-3, CTLA-4, TIGIT, CD160) on peripheral blood mononuclear cells from study participants who were enrolled in the Early Pediatric Initiation, Canada Child Cure Cohort (EPIC[4]) Study. HIV reservoir size was estimated using real-time PCR (total HIV DNA) and the prostratin stimulation assay (inducible HIV RNA).
Results: Study participants ($n = 65$; median age = 14.82 years) were stratified based on age at sample collection (0 - <10 years, n = 14; 10 - <18, n = 34; 18 - 26, n = 17). All participants were on antiretroviral therapy, of whom 84.6% were fully suppressed at the time of sample collection (<40 HIV-1 RNA copies per ml plasma), and those who were not had a maximal viral load of 8473 HIV-1 RNA copies per ml plasma. PD-1, TIGIT and CD160 were the most frequently expressed inhibitory receptors, with frequencies of expression increasing with age and varying across different naïve-memory-effector T cell subsets. Reservoir size in terms of total HIV

DNA and inducible HIV RNA was positively correlated with the frequency of $CD4^+$ T cells expressing PD-1 ($p = 0.013$ and $p = 0.033$, respectively) or TIGIT ($p = 0.028$ and $p = 0.032$, respectively), and with the frequency of $CD8^+$ cells expressing PD-1 ($p = 0.000000735$ and $p = 0.00000536$, respectively), TIGIT ($p = 0.035$ and $p = 0.080$, respectively), or CD160 ($p = 0.021$ and $p = 0.007$, respectively).
Conclusion: Compared to $CD4^+$ T cells, significantly higher frequencies of $CD8^+$ T cells expressed inhibitory receptors and expressed higher numbers of different ones. Markers of T cell exhaustion were associated with HIV reservoir size despite suppression of viral replication with antiretroviral therapy. Exhausted $CD8^+$ cytotoxic T cells may fail to eliminate HIV-infected cells, leading to a larger reservoir. Alternatively, a larger reservoir size (seeded at the time of perinatal transmission) may lead to T cell exhaustion. Overall, our findings suggest that expression of inhibitory receptors and T cell exhaustion may influence reservoir maintenance over time in children, adolescents and young adults with vertically-acquired HIV infection.
This research was funded by grant #HIG-133051 from the Canadian Institutes of Heath Research, the Canadian Foundation for HIV Research, and the International AIDS Society.

Adaptive NK Cell Biology and HIV Control

R. Keith Reeves[1,2,3]
[1]Division of Innate and Comparative Immunology, Center for Human Systems Immunology, Duke University School of Medicine, Durham, NC
[2]Department of Surgery, Duke University School of Medicine, Durham, NC
[3]Department of Pathology, Duke University School of Medicine, Durham, NC

Natural killer (NK) cells provide rapid early responses to viral infections and thus can contribute substantially to disease modulation and potentially vaccine efficacy. Traditionally, NK cells have been considered to be nonspecific components of innate immunity, but burgeoning evidence suggests that the functional repertoire of NK cells is far more diverse and can include adaptive features and memory recall. Some of the first evidence that NK cells respond in an antigen-specific fashion came from experiments revealing that subpopulations of murine NK cells could respond to a specific MCMV protein, and that in the absence of T and B cells, murine NK cells also mediated adaptive immune responses to a secondary challenge with specific haptens. These data have been followed by demonstrations of NK cell memory to viruses and viral antigens in mice, non-human primates, and most recently humans. Indeed recent work from our laboratory and others has shown that adaptive NK cells are mounted against both HIV and SIV antigens, both by infection and multiple vaccine vectors. These responses have proven to be robust, long-lived, and particularly enriched in tissues. Mechanistically, adaptive NK cell responses in humans and non-human primates largely depend on NKG2C expression and MHC-I-mediated presentation on target cells. Further, we have shown at the single cell level antigen-specific anti-HIV NK cell responses are largely dominated by single Env or Gag peptides. These responses are also higher in magnitude in Elite Controllers and can be modulated by therapeutic vaccination. In this presentation a current state of the field will be discussed, including multiple types of memory NK cells, how each type may mobilize against HIV and SIV infection, and how these novel phenomena could ultimately be harnessed in the context of effective cure and vaccine strategies.
Supported by NIH grants R01AI143457, R01AI161010, P01AI162242, UM1AI164570

Engineering B Cells to Control HIV

Geoffrey L. Rogers, Chun Huang, Jason Chang, Xiaoli Huang, Atishay Mathur, Camille Hsu-Yu Chen and Paula M. Cannon
Department of Molecular Microbiology and Immunology, Keck School of Medicine of the University of Southern California, Los Angeles, CA

We use CRISPR/Cas9 gene editing of the immunoglobulin (Ig) locus to reprogram B cells to express custom antibodies. These include broadly neutralizing antibodies that could control HIV *in vivo*. We use a simplified editing protocol based on the design of the heavy chain only antibodies (HCAbs) found in Camelids. By specifically engineering the Ig locus, we preserve important features of natural antibody expression and B cell biology, including response to antigen and differentiation towards antibody-secreting plasma cells.

The HCAb platform is highly flexible. It can accommodate diverse antigen-recognition modules that target HIV gp120, including scFvs, nanobodies, and non-antibody molecules such as CD4 domains. Similarly, the Fc sequence of the HCAb can be optimized to enhance antibody half-life or effector functions, including ADCC killing of HIV-infected cells by NK cells.

To assess the ability of engineered B cells to control HIV, we are engineering both human and macaque B cells with a panel of broadly neutralizing molecules. We confirm the function of the engineered human B cells using tonsil organoids and gp120 vaccination. Our goal is engineer B cells to express a panel of anti-HIV molecules with enhanced breadth and potency, that respond to gp120 vaccination after transplantation, and could thereby provide long-lasting control of HIV.

Single Virus Imaging of HIV-1 Nuclear Import and its Impact on Integration Site Selection

Wout Hannes, Anayat Bhat, Ivan Nombela, Barbara Van Remoortel, Paulien Van de Velde, Frauke Christ, Zeger Debyser
Laboratory for Molecular Virology and Gene Therapy, Department of Pharmaceutical and Pharmacological Sciences, KU Leuven, Leuven, Flanders, Belgium

Background: The mechanism of the early steps of HIV-1 replication including capsid uncoating, nuclear import, and integration remain poorly understood. In particular, the intracellular localization and timing of capsid uncoating is a matter of debate. Moreover, evidence is emerging that nuclear import and integration sites affect the transcriptional state of the provirus. Viral infection is essentially heterogeneous as only a few particles yield an integrated provirus. Conventional molecular biology techniques provide a limited understanding of the process at the ensemble level. Advanced microscopy methods offer the advantage to study the mechanism of viral replication at single virus level.
Aims: Among multiple host factors involved in the HIV-1 infection of host cells, Transportin-SR2 (TRN-SR2), Transportin-1 (TNPO1), and Cleavage and polyadenylation specificity factor subunit 6 (CPSF6) are three host factors that have been implicated in nuclear import of HIV-1. However, the exact mechanism of nuclear import is still under debate. We have established an in-house imaging platform that allows to gain insight in the

mechanism of these host factors in the process of nuclear import. Additionally, understanding the impact of nuclear import on integration site selection and HIV-1 transcription may lead to the identification of new targets for a block-and-lock functional cure.

Methods: To study the role of the host factors TRN-SR2, CPSF6, and TNPO1 in nuclear import, cell lines depleted for each of these host factors were generated. Both single and multiple round infection assays were used to assess the effect of these host factors in HIV-1 infection using a luciferase reporter assay and p24 ELISA respectively. With the established imaging platform, using fluorescently labelled integrase and time-lapse imaging, different steps of the HIV-1 replication cycle in living cells were investigated on a single-particle level. After investigating the effect of these host factors on nuclear import, their impact on integration site selection was studied with integration site sequencing performed on cell lines depleted for each of these host factors.

Results: Nuclear import takes place after transient docking of the viral particles at the nuclear membrane for three to four minutes. In TNPO1-, TRN-SR2-, and CPSF6-depleted cell lines, a 3- to 5-fold increase in the docking time and nuclear import time of the preintegration complexes (PICs) was observed, confirming their role in nuclear import. Whereas depletion of TRN-SR2 hampered single round infection by 60 %, the decrease in single round infection in cell lines depleted for TNPO1 was not significant pointing to a possible redundancy of the host factor in the process of nuclear import. Despite a significant increase in single round infection in cells depleted for CPSF6, a strong reduction was seen in HIV-1 replication in TRN-SR2- and CPSF6-depleted cells in multiple round infection. When sequencing integration sites, a significant reduction in the number of unique integration sites was observed in TRN-SR2-depleted cells. In cell lines depleted for TRN-SR2 and TNPO1, integration was favored in gene bodies and gene dense regions. A significant increase in integration in Refseq genes was seen in TRN-SR2-depleted cells. Moreover, integration was retargeted into chromatin regions with epigenetic marks associated with increased transcriptional activity in these cells (H3K27 acetylation and H3K4 methylation).

Conclusions: We have shown that TRN-SR2, TNPO1, and CPSF6 all have an impact on the process of nuclear import of HIV-1. Despite different effects on single or multiple round infection, nuclear import is delayed when cells are depleted for TRN-SR2, TNPO1, or CPSF6. Moreover, depletion of host factors implicated in nuclear import affects integration site selection. Experiments are planned to investigate the impact on the transcriptional activity of the residual provirus.

This research was funded by the Research Foundation Flanders (FWO) (G091522N and 1S41123N) and the KU Leuven Research Council (C14/17/095–3M170311).

Immunometabolic Remission for Persistent HIV

Ujjwal Neogi[1]

[1]The Systems Virology Lab, Karolinska Institute, Stockholm, Sweden

Background: Viruses are obligatory parasites. The replication and production of the virus is an energy-demanding process that requires both host production of macromolecules and viral hijacking of the host's biosynthesis machinery. The central host metabolic fluxes in the virus-infected cells that affect the viral production have been linked to flux balance in the central carbon and energy metabolism and changes in the lipid biosynthesis pathways. During HIV-1 Infection, the cell's metabolic activity with glycolytic enzymes and activation stage regulates susceptibility to HIV-1, where elevated oxidative phosphorylation (OXPHOS) and

glycolysis favor Infection in CD4+ T cells. A recent study indicated that latent HIV-1 reservoirs use glutaminolysis as an alternative fuel source to generate energy. A minority group of people living with HIV (PWH), called elite controllers (EC), maintains undetectable plasma viremia and low HIV reservoir without antiretroviral therapy (ART). When the ECs lose their elite control status, substantial changes in the metabolic profile were reported, characterized by aerobic glycolytic metabolism, deregulated mitochondrial function, and oxidative stress.
Aim: My group performed extensive clinical and translational studies to identify the critical differences between EC and PWH with long-term ART and identified potential immunometabolic mechanisms of EC status.
Methods: We have developed genome-scale metabolic models (GEM) by integrating multi-omics data both at bulk and single-cell level and identified robust druggable targets using metabolic networks to reduce HIV reservoirs.
Results: The significant system-level cellular and metabolic rewiring in EC occurred around the central carbon and energy metabolism linked to OXPHOS and intermediates of the tricarboxylic (TCA cycle) related to glycolysis and glutaminolysis. More importantly, we observed unique immune-metabolic properties of monocytic/macrophage lineages related to HIV persistence. The modulation of cellular glutaminolysis promoted increased cell death and latency reversal in pre-monocytic HIV-1 latent cell model U1 but not the lymphocytic cell model, indicating that the alteration in the glutaminolysis may be essential for the clearance of the inducible reservoir in HIV-integrated cells.
Conclusion: In the present talk, I will show the application of the GEM to identify the drug target in different cell populations that can provide immunometabolic targets for HIV remission.
The study is funded by the Swedish Research Council grants 2017-01330, 2018-06156, and 2021-01756

The acyl-CoA-binding protein influences autophagy and immune function in people living with HIV

Stéphane Isnard[1,2,3], Léna Royston[1,2,3,4], Tsoarello Mabanga[1,2], Simeng Bu[1,2], Carolina Berini[1,2], John Lin[1,2], Brandon Fombuena[1,2], Nicole Bernard[1,5,6], Guido Kroemer[7,8,9], Jean-Pierre Routy[1,2,10].
[1]Infectious Disease and Immunity in Global Health Program, Research Institute of McGill University Health Centre, Montreal, QC, Canada
[2]Chronic Viral Illness Service, McGill University Health Centre, Montreal, QC, Canada
[3]CIHR Canadian HIV Trials Network, Vancouver, BC, Canada
[4]Division of Infectious Diseases, Geneva University Hospitals, Switzerland
[5]Division of Experimental Medicine, McGill University, Montreal, QC, Canada.
[6]Division of Clinical Immunology, McGill University Health Centre, Montreal, QC, Canada.
[7]Centre de Recherche des Cordeliers, Equipe labellisée par la Ligue contre le cancer, Université de Paris, Sorbonne Université, Inserm U1138, Institut Universitaire de France, Paris, France
[8]Metabolomics and Cell Biology Platforms, Institut Gustave Roussy, Villejuif, France
[9]Institut du Cancer Paris CARPEM, Department of Biology, Hôpital Européen Georges Pompidou, AP-HP, Paris, France
[10]Division of Hematology, McGill University Health Centre, Montreal, QC, Canada

Background: HIV elite controllers (ECs) as compared to people living with HIV (PLWH) receiving

antiretroviral therapy (ART), present with activated autophagy and low glycolysis allowing the establishment and maintenance of efficient anti-HIV T-cell responses. Through degradation of cytosolic structure, autophagy favors the production of IL-21 by TFH CD4 cells which, in turn stimulates efficient anti-HIV CD8 T-cell responses.
The acyl CoA binding protein (ACBP) is a newly identified “autophagy checkpoint”. 1) Cytosolic ACBP binds to activated fatty acid and favors autophagy and oxidative phosphorylation. 2) However, when ACBP is secreted in the extracellular milieu, autophagy is inhibited.
To assess the role of ACBP in PLWH receiving ART, we assessed intra and extracellular ACBP levels and their link with metabolic and immune functions.
Methods: ELISA were used to quantify ACBP and cytokines levels in 50 long-term ART-treated PLWH, 37 ECs and 30 controls without HIV. Intracellular ACBP and autophagy marker LC3II levels were assessed by flow cytometry in PBMC. Metabolomic analyses were performed on serum samples by GC-MS. *In vitro* assays were performed on PBMC from donors without HIV.
Results: ACBP levels were higher in ART-treated PLWH compared to ECs or controls (medians 265, 110 and 121 ng/mL, respectively $p<0.01$ for both comparisons), independently of age and sex. Intracellular ACBP was detected in all leukocytes all groups, and intracellular ACBP levels in T-cells and monocytes inversely correlated with its plasma levels ($r=-0.9$, $p=0.02$ and $r=-0.9$, $p=0.08$, respectively). In ART-treated PLWH, observation showed that intracellular levels of ACBP and LC3II correlated, in CD4, CD8 T-cells and in classical monocytes.
In ART-treated PLWH, plasma ACBP levels were neither associated with CD4 nor CD8 T-cell counts. However, plasma ACBP levels negatively correlated with CD4/CD8 ratio (r-0.34, $p<0.01$) and was positively associated with levels of pro-inflammatory cytokines (IFNα2, IFNγ, IL1β) and homeostatic factors (IL7 and IL15) ($r>0.3$, $p<0.05$ for all comparisons). Plasma ACBP levels were inversely associated with plasma IL-21 levels ($r=-0.54$, $p<0.01$).
PLWH with high plasma ACBP had two-fold higher levels of glutamic acid ($p=0.02$) and tended to have higher levels of α-ketoglutarate ($p=0.09$) in their serum.
In vitro addition of recombinant ACBP at 10µg/mL to culture medium of HIV-negative donors PBMCs for 24h did not change cell viability. However, recombinant ACBP treatment strongly decreased LC3II intracellular levels and the percentage of IFN-γ, IL-2, TNF-α and IL-21 producing CD4 T-cells after PMA+ionomycine stimulation or anti-CD3 ($p<0.05$ for all).
Conclusion: Higher plasma levels of ACBP in ART-treated PLWH were associated with inflammation, elevated glutamic acid and markers of T-cell dysfunction. Circulating ACBP directly weaken anti-HIV T-cell functions, mainly through the inhibition of IL-21 production. Inhibition of circulating ACBP should be considered as a new target for increasing autophagy and improving anti-HIV T-cell responses in a context of cure strategies.

Modeling Sanctuary Site Behavior in the Lymph Node

Ryan Zurakowski [1], Sasan Paryad-Zanjani [1] , Yasaman Moghadamnia [1] , Sobia Anam [1] , Michael Donzanti [1] , Michael J. Piovoso [2] , Jason P. Gleghorn [1]
[1] Department of Biomedical Engineering, University of Delaware, Newark, DE
Department of Electrical and Computer Engineering, University of Delaware, Newark, DE

Background: The lymph node has been implicated as a potential site of ongoing HIV replication in treated persons living with HIV. Various studies have shown reduced concentrations of certain antiretrovirals (ARVs) within the lymph node parenchyma (or lobule), and some of these studies have shown elevated levels of HIV RNA associated with the regions of reduced ARV.
Aims: In order to further investigate the hypothesis that lymph nodes may act as a sanctuary site in treated HIV infection, we have developed a predictive computational model of ARV and HIV dynamics within the lymph node. This model is computed on the reconstructed 3D geometry of real lymph nodes.
Methods: Lymph nodes are sectioned, labeled, and imaged using high-resolution confocal microscopy. The 2D image stacks are reconstructed into a 3D model including the locations of lymph node lobule, sinus, and blood vessels. Extracellular and intracellular transport rates for ARVs are derived both within the lymph node as well as in the blood and fluid lymph. Transport rates for infected and uninfected T cells as well as free HIV virus are also computed. Transport rates for all species between the lymph node, blood, and fluid lymph are also estimated. In all domains (blood, fluid lymph, and lobule), the pharmokinetics of the ARVs and the infection dynamics of HIV are modeled.
Results: Using this model, we are able to replicate the spatial patterns of ARV distribution seen in MALDI imaging of rhesus macaque lymph nodes. Further experiments are underway to identify the transport parameters for several HIV drugs to refine these predictions. We have also shown that the lymph nodes are sufficiently isolated from the blood and fluid lymph to allow for sustained HIV replication even when HIV is suppressed systemically, assuming very low ARV efficacy within the lymph node. Under these circumstances, very few infected cells or virions exit the lymph nodes, so the ongoing replication is predicted to be undetectable from blood measurements. Further work is necessary to determine whether the conditions necessary for this behavior in the computational model are seen in treated persons living with HIV. We are currently working on several extensions to the computational model, including follicular dendritic cell trafficking and antiviral immune responses.
This research was partially supported by the National Institute of Allergy And Infectious Diseases of the National Institutes of Health under Award Number R01AI165368. The content is solely the responsibility of the authors and does not necessarily represent the official views of the National Institutes of Health.

An *In-Vitro* Model Utilizing Fibroblastic Reticular Cells Reveals a Role for AST in HIV Latency

Ashley M Ginda[1], Junia I Alsinawi[1], Rui Li[2], Fabio Romerio[2], Una O'Doherty[1]
[1] Department of Pathology and Laboratory Medicine, University of Pennsylvania, Philadelphia, PA
[2] Department of Molecular and Comparative Pathobiology, Johns Hopkins University School of Medicine, Baltimore, MD

Introduction: Physiologic *in vitro* models of HIV infection represent a critical tool to investigate mechanisms of latency and to evaluate new therapies. While T cell half-lives can be as long as 7 years *in vivo*, primary unstimulated T cell survive less than 14 days *ex vivo*. While the shortened time frame of *ex vivo* culture systems offer an advantage to experimentalists studying HIV infected T cell biology, achieving long term, physiologic culture conditions is important. Fibroblastic reticular stromal cells (FRCs) improve T cell survival when added to culture systems while maintaining cellular phenotype[1]. In addition, many of these latency models utilize fluorescent protein reporter viruses to monitor infection levels over time. A common viral vector (NLENG1-

IRES) was derived from NL43 and inserts a green fluorescent protein (GFP) and IRES between the open reading frames of *env* and *nef* in the HIV genome[2]. This insertion disrupts the HIV antisense transcript (AST). Prior studies show that inhibition of AST results in increased viral transcription associated with epigenetic changes. Thus, AST likely promotes HIV latency by silencing HIV transcription by interacting with the 5' LTR[3,4]. We reasoned that reporter virus that disrupts AST had the potential to impact HIV latency in *ex vivo* culture systems.

Methods: CD4+ T cells were isolated from peripheral blood mononuclear cells from an uninfected donor using magnetic bead separation (EasySep, StemCell Technologies). The isolated CD4+ T cells were infected with either NLENG1-IRES[2] or NL43 using spinoculation[5]. After infection, cells were cultured with and without fibroblastic reticular cells at 10e6/mL in 48 well plates in R30 (RPMI + 30% FBS + 1% penicillin/streptomycin) with saquinavir to allow for one round of replication. Saquinavir was dosed during the culture to prevent ongoing replication. Cells were collected for DNA and flow cytometric analysis on days 3, 6, 10, 12, and 16. Absolute cell counts were obtained to track cell loss and cells were stained for T cell subsets using CD45RA, CCR7, and CD27 and intracellular gag.

Results: Gag expression was similar on day 3 between NLENG-IRES and NL43. The half-life of gag expression in NLENG-IRES infected cells (2.3-3.2 days) is slower than wild-type NL43 infected cells (1.32-1.33 days). The difference between NL43 and NLENG-IRES was greater when cultured on FRCs, while the half-life of gag expression was the same for NLENG-IRES, it was faster still for NL43 (0.74 days).

Conclusions: While it is convenient to use fluorescent reporter viruses to monitor levels of HIV expression in *ex vivo* and *in vitro* culture systems, disruption of AST can change viral dynamics by enhancing HIV expression.

1. Knoblich, K., S. Cruz Migoni, S. M. Siew, E. Jinks, B. Kaul, H. C. Jeffery, A. T. Baker, M. Suliman, K. Vrzalikova, H. Mehenna, P. G. Murray, F. Barone, Y. H. Oo, P. N. Newsome, G. Hirschfield, D. Kelly, S. P. Lee, B. Parekkadan, S. J. Turley, and A. L. Fletcher. 2018. The human lymph node microenvironment unilaterally regulates T-cell activation and differentiation. PLoS Biol 16:e2005046. PMID:30180168. PMC6122729 f. https://www.ncbi.nlm.nih.gov/pubmed/30180168

2. Trinite, B., E. C. Ohlson, I. Voznesensky, S. P. Rana, C. N. Chan, S. Mahajan, J. Alster, S. A. Burke, D. Wodarz, and D. N. Levy. 2013. An HIV-1 replication pathway utilizing reverse transcription products that fail to integrate. J Virol 87:12701. PMID:24049167. PMC3838139. https://www.ncbi.nlm.nih.gov/pubmed/24049167

3. Zapata, J. C., F. Campilongo, R. A. Barclay, C. DeMarino, M. D. Iglesias-Ussel, F. Kashanchi, and F. Romerio. 2017. The Human Immunodeficiency Virus 1 ASP RNA promotes viral latency by recruiting the Polycomb Repressor Complex 2 and promoting nucleosome assembly. Virology 506:34. PMID:28340355. PMC5505171. https://www.ncbi.nlm.nih.gov/pubmed/28340355

4. Li, R., R. Sklutuis, J. L. Groebner, and F. Romerio. 2021. HIV-1 Natural Antisense Transcription and Its Role in Viral Persistence. Viruses 13. PMID:33946840. PMC8145503. https://www.ncbi.nlm.nih.gov/pubmed/33946840

5. O'Doherty, U., W. J. Swiggard, and M. H. Malim. 2000. Human immunodeficiency virus type 1 spinoculation enhances infection through virus binding. J Virol 74:10074. PMID:11024136. PMC102046. https://www.ncbi.nlm.nih.gov/pubmed/11024136

Role of inflammation in Cardiovascular Disease: Lessons learned from HIV and Long COVID

Hsue PY[1,2], Peluso MJ[1,3], Durstenfeld MS[1,2], Henrich TJ[4], and Deeks SG[1,3]
[1]Department of Medicine University of California, San Francisco, CA
[2]Division of Cardiology Zuckerberg San Francisco General, San Francisco, CA
[3]Division of HIV, Infectious Diseases, and Global Medicine, Zuckerberg San Francisco General Hospital University of California, San Francisco, CA
[4]Department of Experimental Medicine University of California San Francisco, CA

Introduction: Persons with HIV (PWH) have an increased risk of cardiovascular disease that includes myocardial infarction, heart failure, and sudden cardiac death. While toxicity from antiretroviral medication and traditional risk factors are important contributors, chronic inflammation/immune activation that persists in the setting of treated suppressed HIV has emerged as a key driver of this disease process. Insights into the role of inflammation and cardiovascular disease have also been elucidated in the setting of the SARS-COV-2 infection and in particular, among individuals who have persistent symptoms following acute infection (termed Long COVID). Our group and others have demonstrated the relationship between inflammation and viral infection (HIV and Long COVID), which in part may be driven by viral persistence leading to immunologic abnormalities.

Methods: A cohort of 152 treated and suppressed persons PWH were studied. Atherosclerosis was assessed using high-resolution ultrasonography of carotid artery intima media thickness at baseline and followup after a mean of 4.2 years. Cell-associated HIV RNA and DNA were measured. Sixty participants after SARS-CO-V-2 infection with and without symptoms underwent cardiopulmonary exercise testing to assess exercise capacity and changes in the heart rate. A cohort of 24 participants after acute SARS-CoV-2 infection was imaged using [18F]F-AraG, which is a selective tracer for activated T lymphocytes. A subset of individuals with Long COVID underwent gut biopsy for evaluation of SARS-CoV-2 RNA.

Results: In the HIV cohort, traditional risk factors and higher IL-6 were associated with higher baseline IMT while viral persistence measures were not. HIV RNA (incidence risk ratio 3.05 [95%CI 1.49-6.27] per IQR, p=0.002) and HIV DNA (IRR, 3.15 [95%CI, 1.51-6.57] per IQR, p=0.002) were significantly associated with incident plaque which remained significant after adjustment for demographics, traditional risk factors and HIV-related factors. Inflammatory markers were associated with higher cell-associated HIV RNA and DNA. In the SARS-CoV-2 cohort, long COVID symptoms were associated with reduced exercise capacity and chronotropic incompetence more than 1 year following acute infection. Higher levels of IL-6 were associated with both abnormalities. T cell activation update was increased even after 2.5 years following acute infection and higher tracer uptake was associated with persistent symptoms. Cellular SARS-CoV-2 RNA was detected in gut tissue in participants (158 to 676 days after acute illness).

Conclusion: Studies of HIV and more recently after SARS-CoV-2 infection continue to play a role in delineating the impact of viral infection and immune activation on cardiovascular disease and persistent cardiopulmonary symptoms. A model is emerging in which viral infection leads to persistence in tissues along with systemic immune responses, with clinical sequelae of symptoms and cardiovascular disease. As inflammation may mediate both of these disease processes, curative strategies in the setting of HIV or Long COVID may be the best therapeutic approach to reduce cardiovascular disease and symptoms. However, in the absence of cure, identification of anti-inflammatory or immunomodulatory strategies are also being investigated.

Reprogramming Resting CD4+ T Cell Metabolism Promotes Proviral Gene Expression and Virions Production From HIV Reservoirs

De Masson d'Autume V[1], LeDouce V[1], O'Reilly S[1], Francois N[1], Angieladis M[1], McGinty T [2], Valle-Casuso JC [3], Macken A [2], Rohr O [5], Van Lint C [6], Sáez-Cirión A [4], Cotter A[2], Feeney E[3], Mallon PW [1,2,3], Gautier VW[1]
[1]UCD Centre for Experimental Pathogen Host Research, School of Medicine, University College Dublin, Ireland
[2] Mater Misericordiae University Hospital, Dublin, Ireland
[3] St Vincent University Hospital, Dublin, Ireland
[4]Institut Pasteur, Unité Réservoirs viraux et contrôle immunitaire, Paris, France
[5]University of Strasbourg, EA7292, DHPI, Institute of Parasitology and Tropical Pathology, Strasbourg, France
[6]Université Libre de Bruxelles, Service of Molecular Virology, Institute for Molecular Biology and Medicine, Gosselies, Belgium

Background: While antiretroviral therapy (ART) efficiently suppresses HIV viremia below detection levels, HIV can persist in long-lived cellular reservoirs (CD4+ memory T cells) where the proviral genome remains silenced but replication-competent. These constitute the main obstacle to HIV cure. Current HIV latency reversal strategies aim at forcing viral replication in the presence of ART so reactivated reservoirs can be eliminated by boosting immune approaches.
HIV gene expression and virion production rely on both strong cellular glycolytic and OXPHOS activities. The tight relationship between metabolic pathways and HIV replication suggests that metabolic requirements for viral reactivation are not met in HIV reservoirs. Indeed, memory CD4 T cells display unique quiescent metabolism that is intimately linked to their resting state, where glycolysis is limited and OXPHOS sustains their basic metabolic needs. In this context, we propose that targeting key metabolic junctions could re-program T cell metabolism to support HIV latency reversal.
Results: Following the screening of small drug molecules targeting OXPHOS, Glycolysis, FAO, FASN, mTOR, or AMPK in primary CD4+ T cells latently infected with HIV *in vitro*, we identified Mitochondrial Pyruvate Carrier inhibitors (MPCi) for their positive impact on HIV transcription. Subsequently, MPCi were tested in *ex vivo* HIV latency reactivation assays using CD8-depleted PBMCs isolated from PLWH on suppressive ART for their capacity to trigger virion production. MPCi promoted HIV production from 9 out of 17 donors and worked in a synergistic manner when combined with Bryostatin in 5 out of 7 donors. These results strongly suggest that MCPi could act as potential Latency Reversing Agents (LRAs).
Previous studies showed that MPCi have been described to block the entry of pyruvate, a glycolysis product, into the mitochondria, where it fuels the TCA cycle and OXPHOS. Instead, MPCi redirects pyruvate towards lactate formation to enhance glycolysis. To examine the specific impact of MCPi on rCD4 T cells, we analysed their glycolytic and mitochondrial respiratory functions using a panel of metabolic assays. First Oxygen Consumption Rate (OCR) was measured immediately after injection of MCPi revealing a 0.7-fold reduction of both maximal respiration (MR) and Spare Respiration Capacity (SCR) (n=3; Seahorse XF Cell MitoStress Test, Agilent). Conversely, MCPi treatment induced an increase in rCD4 T cells glycolytic capacity as measured by the immediate 1.4-fold upregulation of ECAR (n=4, pH Xtra, Agilent)). The increase in glycolysis was sustained at 24hrs and was associated with a 2-fold increased glucose entry (n=3; 2-NBDG; Thermoscientific). Further analysis at 24 hrs post MPCi treatment revealed a significant increase in ATP production linked to both respiration (1.57-fold) and glycolysis (1.4-fold) (Seahorse XFp ATP Real Time, Agilent). This increase of ATP

produced by the mitochondrial respiration might rely on the flexible capacity of cellular metabolism to fuel the TCA with alternative sources of carbon (Fatty Acid and/or Glutamine) and compensate for the lack of pyruvate entry. To measure the impact of MPCi on mitochondria, we measured the mitochondrial mass (n=3, Mitoview Green, Biotum), the Mitochondrial membrane potential (n=3, Mitotracker Red CMXROS, InvitrogenTM) and the production of Reactive oxygen species (n=3, MitoSOX™, InvitrogenTM). While MPCi did not modulate Mitochondrial Mass or ROS production, we observed a significant 0.5-fold reduction in mitochondria membrane potential. Of note, MCPi did not modulate activation markers expression (CD69, CD25, Biolegend) or cellular viability in resting CD4 T cells.
Conclusions: MCPi capacity to reprogramme rCD4 T cells was characterised by an immediate but transient reduction in mitochondrial respiration associated with a concomitant increase in glycolytic capacity followed by an overall increase in ATP production linked to both respiration and glycolysis. We propose that by rerouting pyruvate fate toward lactate formation, MCPi effectively supports HIV reactivation from latency in persistent reservoirs via increasing glycolysis and associated production of key metabolites such as nucleotides, amino acids and fatty acids, the ultimate building blocks for viral particle formation.
Acknowledgements: We thank all participants to this study among the All-Ireland Infectious Diseases (AIID) Cohort. Funding: Irish Health Research Board and *European Union's Horizon 2020 research and innovation programme* under grant agreement No 691119 — EU4HIVCURE — H2020-MSCA-RISE-2015

HIV-2 Reservoirs and Immune Control

Ana E Sousa, MD, PhD
Instituto de Medicina Molecular João Lobo Antunes, Faculdade de Medicina, Universidade de Lisboa, Portugal

Background: HIV-2 disseminates and establishes generalized viral reservoirs. Nevertheless, viremia is low to undetectable in the absence of antiretroviral therapy throughout HIV-2 disease, even in advanced AIDS. Additionally, HIV-2 infection is associated with persistent pan-immune activation, with a direct correlation between the increase of activation markers and the loss of circulating CD4 T cells. However, the rates of disease progression and CD4 T cell decline are very slow, configuring HIV-2 infection as a naturally-occurring attenuated form of HIV/AIDS.
Aims: Our objective is to unravel the unique viral-host interactions underlying the benign prognosis of HIV-2 infection.
Method(s): We have been taking advantage of the significant prevalence of HIV-2 infection within the autochthonous Portuguese population, due to connections to West Africa, where HIV-2 is endemic. Lymph node and gut biopsies, circulating lymphocyte populations, and in-vitro infection assays were used in parallel to investigate HIV-2 vs HIV-1 immunopathogenesis. RNAscope was combined with ddPCR to evaluate viral reservoirs, and spectral flow cytometry was used for immune profiling.
Results: We found major disruption of lymph node architecture in HIV-2-infected individuals, even when circulating CD4 T cell counts were preserved, as well as evidence of local active HIV-2 replication despite the documented low viremia. Germinal center alterations were particularly marked, in agreement with the changes observed in peripheral blood follicular helper and follicular regulatory CD4 T cell subsets and memory B cells. These findings contrasted with the data from the sigmoid and ileal mucosa, where CD4 T cells and epithelial integrity were found to be preserved in spite of the ongoing gut-associated HIV-2 replication. Our research is

currently focused on the identification of the determinants of the tissue pathology/homeostasis and of the HIV-2 production control.
Conclusions: Overall, our data support active HIV-2 replication in lymphoid tissues in the presence of low to undetectable plasma viral load in untreated individuals. The prolonged course of HIV-2 infection leads to lymph node disruption and marked disorganization of germinal centers, but gut mucosa integrity is maintained, which possibly contributes to the slow progression of HIV-2 disease. Ultimately, HIV-2 investigation provides a unique approach to better understand the immune pathology and find novel targets to manage treated HIV-1-infected individuals.

Mimicking HIV-2 Signaling for Optimal T Cell Priming

Victor Appay
Université de Bordeaux, CNRS UMR 5164, INSERM ERL 1303, ImmunoConcEpT, 33000 Bordeaux, France

Background: $CD8^+$ T cells equipped with a full arsenal of antiviral effector functions are critical for effective immune control of HIV-1. It has nonetheless remained unclear how best to elicit such potent cellular immune responses in the context of immunotherapy or vaccination. HIV-2 has been associated with milder disease manifestations and more commonly elicits functionally replete virus-specific $CD8^+$ T cell responses compared with HIV-1.
b We aimed to learn from this immunological dichotomy and to develop informed strategies that could enhance the induction of robust $CD8^+$ T cell responses against HIV-1.
Methods: We developed an unbiased *in vitro* system to compare the *de novo* induction of antigen-specific $CD8^+$ T cell responses after exposure to HIV-1 or HIV-2. The functional properties of primed $CD8^+$ T cells were assessed using flow cytometry and molecular analyses of gene transcription.
Results: HIV-2 primed functionally optimal antigen-specific $CD8^+$ T cells with enhanced survival properties more effectively than HIV-1. This superior induction process was dependent on type I interferons (IFNs) and could be mimicked via the adjuvant delivery of cyclic GMP-AMP (cGAMP), a known agonist of the stimulator of interferon genes (STING). $CD8^+$ T cells elicited in the presence of cGAMP were polyfunctional and highly sensitive to antigen stimulation, even after priming from people living with HIV-1.
Conclusions: HIV-2 primes $CD8^+$ T cells with potent antiviral functionality by activating the cyclic GMP-AMP synthase (cGAS)/STING pathway, which results in the production of type I IFNs. This process may be amenable to therapeutic development via the use of cGAMP or other STING agonists to bolster $CD8^+$ T cell-mediated immunity against HIV-1.

Host-Mediated Control of HIV Disease Progression: Unveiling Abnormal Cortical Actin Dynamics in HIV-1 Infected Primary CD4+ T Cells

Robert L. Furler O'Brien[1]* and Colin Kovacs[2]
[1]Department of Medicine, Division of Infectious Diseases, Weill Cornell Medicine, New York, NY, USA.
[2]Maple Leaf Medical Clinic, Toronto, ON, Canada.

Background: HIV-1 infection disrupts the cortical actin network, leading to morphological and migratory changes in CD4+ T cells. However, discrepancies in reported findings on T cell polarization and migration in

response to HIV-1 infection arise due to various factors, including cell type, environmental complexity, and observation time scales. The role of actin disruption and subsequent cell polarization and migration defects in HIV-1 disease progression is undefined.

Aims: This study aims to unify the disparate findings on HIV-1 induced morphological changes in primary human CD4$^+$ T cells by investigating the progressive morphological differences caused by virally induced cortical cytoskeleton disruption. Additionally, we seek to compare actin disruption in HIV-1 normal progressors and controllers.

Methods: We utilized time-lapse and ultrastructural microscopy to observe HIV-1 infection in primary CD4$^+$ T cells. We compared cells infected with wild-type HIV-1 and Nef-deleted (ΔNef) virus to understand the role of Nef in the observed morphological differences. Furthermore, we investigated the link between HIV-1-mediated actin disruption and diseases affecting the ARP2/3 complex, an essential component of cell migration.

Results: HIV-1 infection in primary CD4$^+$ T cells caused at least five progressive morphological differences, including reduced F-actin content, aberrant cell polarization, decreased migration velocity and directionality, which may lead to compromised endothelial barrier traversal. Of the five aberrant phenotypes, we identified two pathological morphologies unique to HIV-1 infected CD4$^+$ T cells. The observed morphologies mirror those seen in migrating cells containing genetic determinants of primary immunodeficiencies affecting the actin cytoskeleton, particularly diseases involving the ARP2/3 complex. Nef-deleted virus partially ameliorated the dysfunctional phenotype in infected cells. In contrast to all normal progressors and healthy controls, two out of the three HIV-1 controllers tested retained cortical actin stability in the presence of WT infection.

Conclusions: Our findings suggest that HIV-1-mediated actin disruption mirrors ARP2/3 inhibition, leading to distinct pathological morphologies in infected CD4$^+$ T cells. The link between these cellular pathologies and disease progression in normal progressors provides insights into HIV-1 induced immune dysfunction. Additionally, we observed that certain HIV-1 controllers lack these cellular pathologies, possibly indicating a protective mechanism against viral replication and immune response. Restoring ARP2/3 function and cortical actin integrity in HIV-1-infected individuals may present a promising avenue for eradicating infected cells from the body.

Keywords: HIV-1, actin disruption, CD4$^+$ T cells, migration, disease progression, ARP2/3 complex, Nef protein, viral control, immune dysfunction.

Need for ART While Controlling Infection

Nicolas Noel[1,2]
[1] Paris-Saclay University, INSERM/CEA, AP-HP, UMR 1184 Immunology of viral, bacterial, autoimmune and haematological diseases (IMVA-HB), Le Kremlin-Bicêtre, France
[2] AP-HP Paris Saclay, Department of Internal medicine and clinical immunology, Le Kremlin-Bicêtre, France

The term "HIV controller" refers to the small proportion of individuals infected with HIV who can spontaneously control viraemia to maintain very low viral loads (< 1%). One major unresolved question is whether HIV controllers should receive antiretroviral therapy, given that international guidelines recommend treatment for all individuals who are infected with HIV.

Differences in the definitions of a controller (in terms of the viral-load cutoff and the duration of viral control) and contrasting reports on CD4 T-cell decline, chronic immune activation, the cardiovascular risk, and loss of

viral control in controllers have prevented the development of a consensus view. Whether these HIV controllers (HIC) are at risk of HIV-associated comorbidities and could benefit from ART is debated, but recent studies reported decreased T-cell activation upon ART initiation.

The risk of non-AIDS comorbidities possibly related to traditional risk factors (cardiovascular risk, smoking, hepatitis B and C co-infections, ageing...) is being increasingly recognized in HIV controllers. There is a particular attention regarding mental health in people living with HIV/AIDS (including controllers), but the link with immunovirological factors or immune activation has not been proven in spontaneous controllers. While the benefit of classical ART introduction in viremic controllers or patients with recurrent blips seems clear, the need and the modality of therapeutic interventions are matter of discussion in patients with permanent undetectable viral loads and very low-to-undetectable levels of HIV reservoirs.

Reduction of HIV-1 integrated proviral reservoir in PLWH treated with dasatinib and ART: Two case reports

Mario Manzanares[1,2], Guiomar Casado-Fernández[1,3], Montserrat Torres[1,4], Elena Mateos[1,4], Vicente Planelles[5], Christoph Wyen[6], Christian Hoffmann[7], Mayte Coiras[1,4]
[1]Immunopathology Unit, National Center of Microbiology, Instituto de Salud Carlos III, Majadahonda, Madrid, Spain.
[2]PhD Program in Biomedical Sciences and Public Health, Universidad Nacional de Educación a Distancia (UNED), Madrid, Spain.
[3]Faculty of Sciences, Universidad de Alcalá, Madrid, Spain.
[4]Biomedical Research Center Network in Infectious Diseases (CIBERINFEC), Instituto de Salud Carlos III, Majadahonda, Madrid, Spain.
[5]Division of Microbiology and Immunology, Department of Pathology, University of Utah School of Medicine, Salt Lake City, Utah, USA
[6]Department of Medicine I, University Hospital of Cologne, Cologne, Germany
[7]ICH Study Center, Hamburg, Germany

Background: Two PLWH with chronic myeloid leukemia (CML) who were on treatment with antiretrovirals (ART) and dasatinib were followed-up for 4 years to evaluate the effect of lengthy treatment with dasatinib on the HIV-1 reservoir.
Methods: Two Caucasian males with HIV-1 infection and CML were recruited. Blood samples were drawn periodically since 2019. Viral load was analyzed in plasma and integrated provirus was measured in PBMCs by Alu-PCR and nested ddPCR. Provirus reactivation was measured by flow cytometry in CD4 in the presence of brefeldin.
Results: First participant: 1) 50 years-old male diagnosed with HIV-1 infection 17 years ago and CML 13 years ago. He was on BIC/FTC/TAF at the beginning of the study in 2019 and changed to DOL/3TC 4 years ago due to gain weight. He was also on imatinib for 9 years and changed to dasatinib due to fatigue and polyneuropathy. He maintained CML molecular response of 4.0 (BCR::ABL1/ABL1 ≤0.01%) for 3 years 4 months with dasatinib, and during 4.3 months after dasatinib discontinuation. He relapsed of CML and reintroduced dasatinib, which rapidly controlled CML. Main adverse events with dasatinib were fatigue and eczema. HIV-RNA in plasma was undetectable. Median CD4 and CD8 count were 1014 cells/µl (IQR 733-1236) and 639

cells/µl (IQR 442-824), respectively. Median CD4/CD8 ratio was 1.6 (IQR 1.1-1.7). 2) Median log10 PBMCs HIV-1 DNA copies per 10^6 cells was 3.24 during treatment with imatinib. Copies decreased to 2.34 after 3.5 months of dasatinib and to 0.35 (IQR -0.3 to 1.4) from 9.5 months of treatment onwards. After dasatinib discontinuation, proviral DNA was -0.2 (IQR -0.3 to 0.5) during treatment-free remission (TFR). After CML relapse and dasatinib reintroduction median log was -0.35 after 3.7 months of treatment.
Second participant: 1) 62 years-old male diagnosed with HIV-1 infection 25 years ago and with CML 4 years ago who was on ART with AZT/RPV/TDF at CML diagnosis and initiated BIC/FTC/TAF in 2019 to avoid interactions with dasatinib as first line. He showed CML molecular response 3.0 (BCR::ABL1/ABL1 ≤0.1%) and undetectable viral load. Median CD4 and CD8 count were 812 cells/µl (IQR 787-1309) and 3251 cells/µl (IQR 2231-3909), respectively. Median CD4/CD8 was 0.3 (IQR 0.2-0.4). 2) Log10 PBMCs HIV-1 DNA copies per 10^6 cells was 1.32 after 5 months of dasatinib and 0.88 (IQR 0.3 to 1.0) since 1 year 4 months onwards. No detectable viral reactivation from isolated CD4 was observed in any participant during treatment with dasatinib.
Conclusions: Periodical treatment of PLWH on ART with dasatinib may be a strategy to progressively reduce HIV-1 reservoir size and control proviral reactivation.

Figure 1. Analysis by ddPCR of changes in HIV-1 reservoir size in two PLWH on ART and dasatinib.

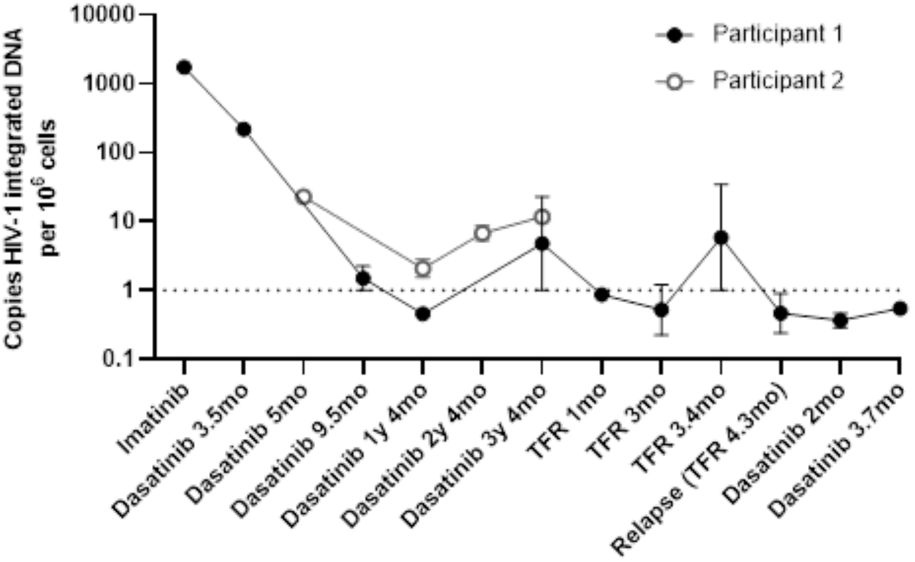

O'Doherty U, Picado JM, Cirion AS. Highlights from the Inaugural HIV Reservoirs and Immune Control Conference, October 1st- 4th 2023, Malahide Ireland. *Pathogens and Immunity*. 2023;8(1):161-169.doi: 10.20411/pai.v8i1.653

Early ART initiated during acute HIV infection has a major impact on naïve CD4+ T-cells

Robert Badura[1,2], Guilherme B. Farias[1], Beatriz Moleirinho[1], Emily Jelagat[1], Margarida Paulo Pedro[1], Diana F. Santos[1], André M.C. Gomes[1], Rita T. Marques[1], Zoe Junginger[1], Carolina M. Conceição[1], Ana Godinho-Santos[1], Afonso R. Almeida[1], Ana E. Sousa[1]
[1]Instituto de Medicina Molecular João Lobo Antunes, Faculdade de Medicina, Universidade de Lisboa;
[2]Hospital de Santa Maria, Centro Hospitalar Universitário Lisboa Norte, Portugal.

Background: The homeostasis of the naïve CD4$^+$ T-cell pool has been emerging as a key target in the context of HIV infection not only for the maintenance of immunoregulation and inflammation but also for the control of the viral reservoir through a dynamic renewal of the memory cell population, including T memory stem cells. In particular, Recent Thymic Emigrants (RTE), defined by high expression of the adhesion molecule CD31 (PECAM-1), are of major interest since they are highly sensible to IL-7-induced homeostatic proliferation, and they have been shown to be preferential precursors for Stem cell Memory (Tscm) and regulatory (Treg) T-cells. Our aim was to compare the naïve CD4$^+$ T-cell compartment in HIV-infected individuals under suppressive antiretroviral therapy (ART), namely of HIV-1+ individuals who started treatment in the early Fiebig stages of acute infection, known to have a less eminent inflammatory state, versus those who initiated ART in advanced chronic phase of disease. We also compared these to individuals infected by HIV-2, which maintain an inflammatory state although associated with an indolent progression of their disease and low to undetectable viremia throughout the natural course of the disease.
Materials and Methods: We take advantage of rare patients under follow-up at our department, namely HIV-1-infected individuals that started ART in very early Fiebig stages (n=16), and compare with HIV-1 infected patients who initiated their treatment in advanced stage of their disease (n=19), individuals infected with HIV-2 (n=18) and age-matched healthy controls (HC, n=21). A panel of cell markers were used to investigate their naïve CD4$^+$ T cells (CD45RO, CCR7, CD31, CD127, Foxp3, CD25, CD95, CXCR3, CD122, CCR6, CCR4, CXCR5, Ki67, CD39, PD-1, HLA-DR) by spectral flow cytometry in parallel with viral parameters (cell-associated pro-viral DNA by digital droplet PCR). For a clear and comprehensive visualization of subpopulations and their respective phenotypes, we applied dimension reduction, generated UMAPS and clustering algorithms. For statistical analysis and comparison between groups we used Mann-Whitney U test and for repeated measures ANOVA test. P values below 0.05 were considered significant.
Results and Conclusions: Early ART initiation resulted in a major recovery of any naïve T-cell compartment alteration, turning them comparable to seronegative healthy controls, without statistical significance differences between all the identified clusters. CXCR3$^+$ naïve T cell subpopulations including a stem cell memory subpopulation, characterized by the expression of PD1$^+$ in addition to CXCR3$^+$ and CD95$^+$, were significantly expanded in both HIV-1 and HIV-2 infected individuals when compared to either HC or early treated HIV-1 individuals, despite the long-term suppressive ART in all HIV+ individuals (8.2±0.9 years). These similarities between HIV-1 and HIV-2 are of interest also as they seem to be independent of previous exposure to high peripheral viral load. Curiously, the main difference found for HIV-2 versus all the other cohorts was a significant expansion of a naïve Treg CD4$^+$ T-cell subpopulation characterized by the expression of CD95$^+$ and CXCR3$^+$, in addition to CD25, Foxp3, and naïve markers, with potential role on inflammatory control and on the slow disease progression known to be the hallmark of HIV-2 infection.
Our findings support an impact of the timing of ART and of the type of HIV infection on the heterogeneity of the naïve CD4 T-cell compartment that deserves deeper functional evaluation to clarify its contribution to

control inflammation and viral reservoirs.

This research was funded by PTDC/MED-IMU/0938/2020 funded by Fundação para a Ciência e a Tecnologia (FCT); PTDC/MED-IMU/30474/2017 cofunded by FCT and FEDER LISBOA-01-0145-FEDER-030474, through Programa Operacional Regional de Lisboa, do PORTUGAL 2020; and Program Gilead Genesis
